# New approach methodologies (NAMs) for the *in vitro* assessment of cleaning products for respiratory irritation: workshop report

**DOI:** 10.3389/ftox.2024.1431790

**Published:** 2024-10-08

**Authors:** Lynne T. Haber, Mark A. Bradley, Amanda N. Buerger, Holger Behrsing, Sabina Burla, Phillip W. Clapp, Scott Dotson, Casey Fisher, Keith R. Genco, Francis H. Kruszewski, Shaun D. McCullough, Kathryn E. Page, Vivek Patel, Nathan Pechacek, Clive Roper, Monita Sharma, Annie M. Jarabek

**Affiliations:** ^1^ Risk Science Center, Department of Environmental and Public Health Sciences, University of Cincinnati, Cincinnati, OH, United States; ^2^ Stantec ChemRisk, Cincinnati, OH, United States; ^3^ Institute for In Vitro Sciences, Inc., Gaithersburg, MD, United States; ^4^ Invitrolize sarl, Belvaux, Luxembourg; ^5^ Wake Forest Institute for Regenerative Medicine, Winston-Salem, NC, United States; ^6^ Insight Exposure and Risk Sciences Group, Cincinnati, OH, United States; ^7^ Ecolab, St. Paul, MN, United States; ^8^ Arkema Inc., King of Prussia, PA, United States; ^9^ American Cleaning Institute®, Washington, DC, United States; ^10^ Public Health and Integrated Toxicology Division, Center for Public Health and Environmental Assessment, Office of Research and Development, U.S. EPA, Chapel Hill, NC, United States; ^11^ The Clorox Company, Pleasanton, CA, United States; ^12^ Roper Toxicology Consulting Limited, Edinburgh, United Kingdom; ^13^ PETA Science Consortium International e.V, Stuttgart, Germany; ^14^ Health and Environmental Effects Assessment Division, Center for Public Health and Environmental Assessment, Office of Research and Development, U.S. EPA, Chapel Hill, NC, United States

**Keywords:** new approach methodologies, cleaning products, respiratory irritation, adverse outcome pathway, air-liquid interface, inhalation dosimetry, *in vitro*, best practices

## Abstract

The use of *in vitro* new approach methodologies (NAMs) to assess respiratory irritation depends on several factors, including the specifics of exposure methods and cell/tissue-based test systems. This topic was examined in the context of human health risk assessment for cleaning products at a 1-day public workshop held on 2 March 2023, organized by the American Cleaning Institute^®^ (ACI). The goals of this workshop were to (1) review *in vitro* NAMs for evaluation of respiratory irritation, (2) examine different perspectives on current challenges and suggested solutions, and (3) publish a manuscript of the proceedings. Targeted sessions focused on exposure methods, *in vitro* cell/tissue test systems, and application to human health risk assessment. The importance of characterization of assays and development of reporting standards was noted throughout the workshop. The exposure methods session emphasized that the appropriate exposure system design depends on the purpose of the assessment. This is particularly important given the many dosimetry and technical considerations affecting relevance and translation of results to human exposure scenarios. Discussion in the *in vitro* cell/tissue test systems session focused on the wide variety of cell systems with varying suitability for evaluating key mechanistic steps, such as molecular initiating events (MIEs) and key events (KEs) likely present in any putative respiratory irritation adverse outcome pathway (AOP). This suggests the opportunity to further develop guidance around *in vitro* cell/tissue test system endpoint selection, assay design, characterization and validation, and analytics that provide information about a given assay’s utility. The session on applications for human health protection emphasized using mechanistic understanding to inform the choice of test systems and integration of NAMs-derived data with other data sources (e.g., physicochemical properties, exposure information, and existing *in vivo* data) as the basis for *in vitro* to *in vivo* extrapolation. In addition, this group noted a need to develop procedures to align NAMs-based points of departure (PODs) and uncertainty factor selection with current human health risk assessment methods, together with consideration of elements unique to *in vitro* data. Current approaches are described and priorities for future characterization of *in vitro* NAMs to assess respiratory irritation are noted.

## 1 Introduction

The American Cleaning Institute^®^ (ACI)^1^ sponsored a workshop in Arlington, Virginia on 2 March 2023, regarding the use of *in vitro* new approach methodologies (NAMs) for the assessment of cleaning products and ingredients for respiratory irritation. To avoid limiting the discussion, NAMs were not defined in the context of the workshop, and definitions may vary across different organizations. A recent definition from the US EPA is “any technologies, methodologies, approaches, or combinations thereof that can be used to provide information on chemical hazard and potential human exposure that can avoid or significantly reduce the use of testing on animals” (U.S. EPA, 2023). Respiratory irritation is one of the leading health concerns associated with the inhalation of chemicals in consumer and workplace scenarios. In a review of the health risks of chemicals in consumer products, Li and Suh (2019) found that 50% of the identified chemicals caused irritation. Similarly, almost 1/3 of the occupational exposure limits (OELs) reviewed by Paustenbach (2000) were based on odor or irritation. Irritation was not defined in the studies by Li and Suh (2019) or Paustenbach (2000), but for the purposes of this workshop, respiratory irritation was defined as disruption of the epithelial lining fluid (ELF) or epithelial perturbation (e.g., disruption of the cell membrane, inflammation, or cytotoxicity). Thus, respiratory irritation can occur throughout the respiratory tract, including in the pulmonary region. Conversely, respiratory sensitization, sensory irritation (i.e.*,* irritation resulting from stimulation of specific nerve receptors), and neurogenic inflammation were not included within the workshop scope.

Animal studies conducted to characterize respiratory responses to potential chemical irritants pose unique technical and scientific challenges and are often high cost and low throughput. To address these concerns, toxicity testing in Organisation for Economic Co-operation and Development (OECD) member countries is being increasingly directed towards systems that can provide data relevant to human biology and mechanisms of toxicity while moving away from animal testing. Key milestones include the European Union (EU) directive limiting cosmetic product testing on animals (European Union, 2003), the associated regulation (European Union, 2009), and the 2007 National Research Council (NRC) report on toxicity testing in the 21^st^ Century (National Research Council, 2007). More recently, a key objective of the Registration, Evaluation, Authorisation and Restriction of Chemicals (REACH) legislation in the EU was to promote non-animal test methods, as exemplified by European Chemicals Agency (ECHA) guidance (European Chemicals Agency, 2011; European Chemicals Agency, 2016). Similarly, the United States (U.S.) Environmental Protection Agency (EPA) released a strategic plan to promote the development and implementation of alternative test methods within the Toxic Substances Control Act (TSCA) program (U.S. EPA, 2018), as well as a workplan for reducing vertebrate animal testing and increasing scientific confidence in and application of alternative methods (U.S. EPA, 2021). These alternative methods, including *in vitro* testing, testing of non-vertebrate organisms, *in silico* modeling, read-across, among others, are collectively termed NAMs and are increasingly being used for both regulatory and non-regulatory internal decision making (Stucki et al., 2022; Lee et al., 2022; Westmoreland et al., 2022; Miller-Holt et al., 2022; Schmeisser et al., 2023).

An important advantage of *in vitro* testing is that it uses cells from the species of interest (i.e., humans), with the potential to allow for the evaluation of inter-individual variability, and for the study of population variability (e.g., children and susceptible populations) where *in vivo* exposure studies may not be ethical. There are, however, additional parameters that need to be considered for the incorporation of *in vitro* NAMs to predict respiratory irritation and for decision making. Limitations of *in vitro* testing for respiratory irritation may include the use of single cell lines or limited cell types to represent a spatially diverse and complex system of at least 41 cell types, limited capacity for long-term exposures, lack of metabolic capacity, inadequate replicates, limited understanding or measurement of internal dose, no consideration of systemic sequelae; as well as the use of systems, assays, and methods that have not been thoroughly optimized and/or characterized. Several of these challenges may be resolved with additional research and modification of test methods and test systems, but some are likely to be inherent to *in vitro* testing. Creating a single *in vitro* test system that contains all cell types found in the respiratory tract is not technically feasible, but the effects on specific regions of the respiratory tract might be predicted with experimental systems containing the cells critical to a given pathogenesis (Clippinger et al., 2018a).

The workshop focused on issues related to cleaning products and their components and was specifically targeted to issues faced by manufacturers and formulators of cleaning products and of their ingredients and intermediates when designing toxicity testing for the intended use of these materials. The organizers defined a cleaning product as any product whose purpose is to remove a “soil”^2^. “Soils” can be complex and variable. Examples of “soils” include grass stains on clothing, scale on shower walls, and biological material on a scalpel. The chemical heterogeneity of soils, together with the diversity of surfaces to be cleaned, necessitates the use of a variety of different types of chemical agents. Addressing cleaning products is a challenge, since cleaning products are typically complex formulations, with each component serving a specific function. Thus, a single cleaning product may be a mixture of surfactants, builders with an array of functions (e.g., anti-corrosion, deflocculation, chelation), solvents, antimicrobials, enzymes, dyes, fragrances, preservatives, and water (ACI, 2024).

Broadly, participants recognized three potential applications of *in vitro* respiratory irritation assays for cleaning product formulations and/or ingredients, with the idea that the test should fit the objective and context of use: (1) qualitative or semi-quantitative screening-level hazard identification, (2) industry qualitative or semi-quantitative risk assessment, and (3) deriving a toxicity reference value (TRV, a generic term for health benchmark values such as a Reference Concentration, RfC) or a quantitative risk assessment conducted for the purposes of regulatory acceptance (Table 1). Industry may utilize qualitative screening-level hazard identification to inform inclusion or exclusion of ingredients in formulations, or to identify product formulation candidates for further development; regulatory bodies may use screening-level hazard and/or exposure assessments to prioritize chemicals for TRV derivation or a full risk assessment. For example, a negative respiratory irritation result in a simpler *in vitro* test may be sufficient to pass hazard screening of an ingredient or cleaning product internally, so that the ingredient/product can undergo further testing in a more complex *in vitro* system. Alternatively, a positive result may result in a decision not to proceed with development or use of that formulation or ingredient.

**TABLE 1:** Cleaning product and ingredient assessment types and uses for industry and regulatory purposes.

	Assessment type and use		
	Screening (qualitative or semi-quantitative)	Intermediate (qualitative or semi-quantitative)	Full (quantitative)
Industry – Internal Activities and Decision Making	Include/exclude ingredient or product candidates	Internal risk assessment for ingredients or product formulations	Submission of full risk assessment for regulatory approval of ingredients and product formulations (if applicable)
Regulatory	Screening/prioritization for full risk assessment (hazard and/or exposure)	N/A	Review of full regulatory risk assessments for ingredients Derivation of TRVs Not applicable to cleaning product mixtures

Key: N/A: not applicable; TRV: toxicity reference value, a term equivalent to health benchmark value.

In industry, internal risk assessments (qualitative or semi-quantitative) can be conducted for product formulations or ingredients. This may be the final risk assessment step for cleaning product formulations, or this step may act as a precursor to the quantitative risk assessment for regulatory purposes for single chemical ingredients used in the cleaning product. For example, under Federal Insecticide, Fungicide and Rodenticide Act (FIFRA)^3^, which regulates cleaning products with antimicrobial claims, there are different regulatory testing requirements for product formulations (which are typically complex mixtures) versus individual ingredients. Specifically, evaluation of antimicrobials under FIFRA requires acute inhalation toxicity studies of the active ingredient and end-use product; a repeated dose (90-day) animal study may be required for the active ingredient(s) but repeat-dose testing is not required for the mixture in the final product (CFR, 2013). Risk is evaluated for new and existing chemicals under the TSCA, and for worker exposure under the Occupational Safety and Health Act (OSHA), but there are no associated minimum data requirements. The FIFRA testing requirements and lack of specific testing requirements under TSCA and OSHA mean that respiratory irritation may not have been evaluated *in vivo* for the cleaning product. Therefore, manufacturers and formulators may use NAMs internally to inform respiratory irritancy and potency of cleaning product formulations that are not captured in *in vivo* tests and not required in regulatory submissions. This internal use of NAMs by manufacturers and formulators was the primary, but not exclusive, focus of the workshop.

The quantitative or final assessment in industry for cleaning product formulations and ingredients may be submitted for regulatory approval, which requires different types of acute and repeated dose toxicity tests, as described above. The regulatory authorities review these quantitative risk assessments or may develop their own risk assessments and derive TRVs. This level of regulatory review and acceptance is not covered in detail in this manuscript, as the intent is to cover evaluation of cleaning products prior to when this type of quantitative assessment is applicable.

Due to the wide variety of cleaning products, chemical classes were not specified as part of defining the scope of the workshop. However, it is acknowledged that certain approaches (exposure systems, calculation methods, *in vitro* cellular or tissue test systems, etc.) are not appropriate, not relevant, or are less desirable for certain chemical classes and/or physicochemical characteristics, and this was noted when appropriate. It was noted that typical liquid aerosol particle sizes generated during cleaning product use would be expected to be >10 µm, and that pulmonary deposition is often considered to be restricted to particles smaller than 10 µm. Thus, it is generally assumed that cleaning products are unlikely to generate particles small enough to reach the pulmonary region of the lung. However, some products/ingredients may increase or reduce particle size prior to reaching the breathing zone. Additionally, all particulate exposures occur with a size distribution, rather than as a single uniform size, and may even be multi-modal, such that an aerosol with a particle median diameter >10 µm could still have a substantial fraction of smaller particles depending on its size distribution. Further, particles of 10–30 µm can deposit in the tracheobronchial region and upper airways. Therefore, consideration of the entire particle size distribution and characterization of the particle size distribution under relevant exposure scenarios is important, especially as it determines the location of deposition in the respiratory tract. As further addressed in Sections 3.1.2, 6.2, the Multi-path Particle Dosimetry (MPPD) model uses particle size distribution in calculating deposited dose.

Delmaar and Bremmer (2009) conducted an extensive investigation of the mass generation rate and particle size distribution of spray cans and trigger sprays, as part of the development of the ConsExpo spray model, which is commonly used for modeling exposures to consumer products. They found that trigger sprays, which use mechanical force, produce larger aerosols than spray cans, which use a pressurized propellant gas. For almost all of the cleaning products tested, 1.3% or less of the total mass sprayed had a particle size ≤10 μm; the sole exception was a spray can product with 9% of the mass having a particle size ≤10 µm. These data support the assumptions described in the previous paragraph, but it is important to ensure that particle size distribution is assessed during product development.

For the purposes of the workshop, exposure scenarios of interest were defined as acute episodic exposures and repeated exposures, relevant to consumer and occupational exposure environments (e.g.*,* workers at commercial cleaning companies). The workshop specifically excluded from its scope issues related to assessment of effects after subchronic or chronic repeat dose inhalation testing, including evaluating systemic effects. Rather, the assays addressed by the workshop would often be used for ranking and screening or creating context regarding effects as part of a weight of evidence (WOE) evaluation for product safety.

The public was invited to the workshop, and specific expert panelists from industry, government organizations, and non-governmental organizations were invited to attend. It is acknowledged that this method of invitation likely did not result in attendance fully representative of worldwide experts. The goals of this workshop were to (1) review *in vitro* cellular and tissue-based NAMs for evaluation of respiratory irritation, (2) examine different perspectives on current challenges and suggested solutions, and (3) publish a manuscript of the proceedings. The aim of these proceedings is to assist the cleaning products industry in best practices and principles (including identification of potential issues and concerns) when selecting testing methods to assess the respiratory irritation potential of their products.

The plenary session included an introduction to adverse outcome pathways (AOPs), as well as an overview of available exposure systems and *in vitro* cell/tissue culture test systems. Section 2 of this manuscript introduces the use of AOPs to inform application of NAMs data. The panel discussions were focused primarily on three-dimensional (3D) transwell insert test systems derived from either primary cells (also known as reconstituted human airways [RHuA]) or cell lines cultured at the air-liquid interface (ALI). Other test systems are available but were not considered in detail by the panelists. These additional test systems include submerged cultures, cell lines (e.g.*,* H292 [muco-epidermoid carcinoma], A549 [alveolar basal epithelial cells], and BEAS-2B [normal human bronchial epithelium, immortalized]), organoids and spheroids, lung on chip systems (often with 3D test systems, cell lines, and organoids incorporated into them), and human precision cut lung slices (hPCLS). Further information on different test systems and their advantages and disadvantages are discussed in other literature (Zavala et al., 2020; Polk et al., 2016; Clippinger et al., 2018b). Specific issues identified include choice of cell/tissue test system and exposure system, and the importance of considering inter-individual variability in the test systems and time course of potential responses.

The initial plenary introduction was followed by three concurrent breakout sessions, on exposure methods, *in vitro* cell/tissue test systems, and application considerations for human health protection. Charge questions for the breakouts are presented in the Supplementary Material. These breakout sessions are summarized in Sections 3, 4, 6, respectively. Section 5 summarizes an illustrative (and not specifically recommended) option for a tiered testing approach that was discussed by the breakout group on *in vitro* cell/tissue test systems. The breakouts were followed by plenary summaries of the breakout sessions and associated discussion.

A key feature of the workshop was to provide the opportunity for sharing diverse perspectives, especially as NAMs represent an emergent technology. Therefore, no attempt was made to reach consensus, although areas of agreement are noted. Where participants disagreed, alternative perspectives are presented in these proceedings. Finally, the focus of these proceedings is to summarize the discussions that occurred *at the workshop.* Some additional explanations have been added for clarity, but these proceedings are not intended to be a comprehensive review of the literature. Where specific examples are listed, this is intended to provide context, rather than being exhaustive.

## 2 Use of adverse outcome pathways to inform application of NAMs data

AOPs play a key role in the use of NAMs in human health risk assessment. An AOP is defined as “a sequence of events commencing with initial interactions of a stressor with a biomolecule in a target cell or tissue (i.e.*,* molecular initiating event [MIE]), progressing though a dependent series of intermediate events, and culminating in an adverse outcome” (OECD, 2017; OECD, 2018b). These intermediate events are termed Key Events (KEs), and the response-response relationship between KEs are termed Key Event Relationships (KERs). An AOP is similar to mode of action (MOA), but the AOP begins with the molecular interaction of the chemical and a target, and does not consider physicochemical properties or absorption, distribution, metabolism and elimination (ADME), which are key determinants of a MOA. By excluding the chemical-specific parts of the MOA, an AOP is “chemically agnostic,” facilitating application of an AOP in a modular fashion to various chemicals and additional stressors (Villeneuve et al., 2014a; Villeneuve et al., 2014b).

An advantage of the AOP conceptual construct is that it provides the structure for designing a testing strategy based on MIE, KEs, and KERs within an AOP, which can then be assessed using *in vitro* methods (Clippinger et al., 2018a; Carusi et al., 2018; Leuttich et al., 2021). A fully defined AOP is not always needed to comprehensively understand likely and important key events of pathogenesis induced by an exposure; instead, testing may be focused on mechanistically characterizing one or a few KEs. It is important to evaluate NAMs data based on mechanistic understanding and based on data from other, well-studied chemicals. An Integrated Approach to Testing and Assessment (IATA) provides a framework for integrating all of the available data and data types in support of the application of NAMs (Worth and Patlewicz, 2016; Kang et al., 2021). An IATA is designed to obtain and combine salient information to allow a decision to be made in the most efficient way, accounting for the context of use. As defined by the U.S. National Toxicology Program^4^, an IATA “provides a means by which all relevant and reliable information about a chemical is used to answer a defined hazard characterization question. Information considered can include toxicity data, computational model predictions, exposure routes, use cases, and production volumes” and may include various AOPs. A defined approach (DA) consists of a selection of information sources (e.g., *in silico* predictions, *in chemico*, *in vitro* data) used in a specific combination, and resulting data are interpreted using a fixed data interpretation procedure (DIP) (e.g., a mathematical, rule-based model). A DA can be used in an IATA or on its own to satisfy the need for hazard information. A DA “can be applied to data … generated with a defined set of information sources to derive a prediction without the need for expert judgment … ” and is intended to overcome some limitations of the individual, stand-alone methods (OECD, 2020; OECD, 2022; OECD, 2023). For example, several DAs were developed to characterize various key events based on an established AOP for skin sensitization (Kleinstreuer et al., 2018).

Although no respiratory irritation AOP has been developed^5^, some efforts have used existing AOPs to inform human health risk assessment for respiratory toxicity, including respiratory irritation. Clippinger et al. (2018a), Clippinger et al. (2018b) described considerations and tools that are useful to develop an IATA for assessing acute inhalation toxicity, informed by KEs in various possible AOPs. Ramanarayanan et al. (2022) described an AOP-based approach to assess respiratory toxicity of a contact irritant using a NAM, which was subsequently developed into an OECD case study (OECD, 2022). Pauluhn (2022) noted the importance of considering respiratory tract region and physicochemical properties in the context of AOP development. Sharma et al. (2023) have developed a case study on *in vitro* systems to assess respiratory toxicity. As discussed further in the rest of these proceedings, establishing confidence in a testing approach is critical for its use in health risk assessment (van der Zalm et al., 2022).

## 3 Exposure methods

Rather than identifying a set of prescriptive standards for the exposure methods to be used in *in vitro* assessments of respiratory irritation, the workshop participants defined key considerations in the selection of appropriate *in vitro* exposure methods based upon the intended application of the data obtained. Such considerations are linked to the purpose and question(s) of interest in the respiratory irritation assessment, as outlined in Table 1. Therefore, it was suggested that exposure methods be selected on a fit-for-purpose basis or for the intended application or context of use (van der Zalm et al., 2022). For example, screening level or prioritization assessments may utilize submerged exposures, whereas assessments for regulatory submissions or contributions to TRV derivation may utilize *in vitro* exposures more relevant to human inhalation exposures. Given the fit-for-purpose nature of selecting *in vitro* exposure methods, documentation of the rationale for the choice of *in vitro* exposure method is needed. Use of inadequate exposure methods or lack of proper documentation of *in vitro* exposure methods can limit the applicability and reliability of the results in the hazard or risk assessment (Whalan et al., 2019; Petersen et al., 2023). Thus, participants emphasized the necessity of establishing reporting standards that adequately document the methods and provide scientific justification for the selection of those methods (van der Zalm et al., 2022). At the time the workshop was held, no *in vitro* reporting guidelines were available; however, the United Kingdom-based National Centre for Replacement, Refinement, & Reduction of Animals in Research has since submitted the Reporting *In Vitro* Experiments Responsibly (RIVER) Recommendations (2023).

### 3.1 Connecting *in vitro* exposure scenario to anticipated or known human exposure

The participants highlighted the importance of the relevant *in vivo* occupational or consumer exposure scenario in selection of *in vitro* delivery method, dose selection, exposure regimen (both duration of exposure and use of recovery periods), and exposure method. However, it was acknowledged that cleaning product or ingredient characteristics must be considered in, and sometimes drive, the *in vitro* exposure design. These considerations include the testing of a single ingredient or the cleaning product mixture, the physicochemical properties of the cleaning product and/or ingredient (including aerosol size distribution), and the product packaging (e.g.*,* spray bottle versus aerosol cannister). Such aspects of the cleaning product or ingredient may inform aspects of the exposure scenario (e.g*.,* mode of delivery based on product form) as they affect the intended use of the product and could impact performance of the assay. This section (Section 3.1) outlines considerations in selection of a relevant *in vitro* exposure based on the pertinent human exposure.

#### 3.1.1 *In Vivo* user exposure scenario

Proper problem formulation of the *in vivo* exposure(s) of interest is foundational in the design of relevant *in vitro* exposure methods. The *in vivo* exposure scenario was discussed in terms of tailoring the *in vitro* system to the exposed populations and their range of expected and potential use scenarios. Broadly, the intended exposure scenarios of cleaning products and/or ingredients were expected to fall into three categories: (1) manufacturers/formulators (i.e., those manufacturing raw ingredients or those combining the raw ingredients into a cleaning product), (2) industrial cleaners (i.e*.,* those using cleaning products under an occupational exposure scenario), and (3) retail consumers (i.e., those using cleaning products residentially). Each of these intended users has a different usage pattern and exposure profile, as well as varying use and types of personal protective equipment (PPE). For example, a worker in a manufacturing scenario may be exposed to a particular ingredient for intermittent periods during 8-hour or 12-hour production days or an industrial cleaner may be exposed to a cleaning product daily for the entire duration of their employment (e.g.*,* 8 h per day for years, leading to a subchronic or chronic exposure), with or without PPE; while a retail consumer may be exposed for a short duration (e.g.*,* 15 min) once per week over months or years with little or no use of PPE. Another categorization of user types that the participants considered was the use of three broad categories of users for the cleaning product or chemical ingredient of interest: average users, high end users, and bystanders. For example, the average retail consumer may use a product once per week, while a retail high end user may use the cleaning product once per day or more; a bystander may be a spouse or child in the home where the cleaning product is used by either an average or superuser. Category definitions of average users, high end users, and bystanders can be established on a case-by-case basis.

In addition to consideration of the *in vivo* exposure scenario through the lens of the anticipated cleaning product or ingredient user, there are additional attributes that need to be considered in the selection of exposure methods for risk assessment of respiratory irritation. Risk assessment has historically assumed (implicitly or explicitly) a 70 kg adult male to be the receptor for hazard identification and risk calculation purposes, whereas current practice is moving toward expanding the life stages, genders, sizes, and other susceptibility factors (e.g., people with asthma; evaluation of different activity levels, which can affect minute volume) to be considered during the assessment process. Concern about potential exposure of susceptible populations to cleaning products or ingredients may affect the frequency and duration of their use in certain settings (e.g., hospitals). Further, assessments should consider not only exposure from the intended use of a product, but also the potential for exposures resulting from unintended or unexpected use of the cleaning product or ingredient, such as an acute high dose exposure from a spill. The pertinent dosing regimen may need to consider both potential for aggregate exposure to the cleaning product and/or ingredient via multiple routes, and cumulative exposure to multiple cleaning products and/or ingredients. The exposure scenario incorporates aspects of not only the duration and the intensity of exposure but may also need to consider recovery times to account for the intermittent use of cleaning products by some users (e.g.*,* retail users). Additionally, the product form and packaging (e.g., spray bottle, pump, aerosol) will impact the use and exposure profile. These challenges related to the user populations and their use scenarios are not unique to *in vitro* models, as the same considerations are made in determining *in vivo* experimental exposure methods.

#### 3.1.2 Determining *in vitro* exposure scenario

The variety of use scenarios described in Section 3.1.1 illustrate the importance of both the intensity and duration of the *in vivo* exposure, which in turn impacts the choice of *in vitro* exposure concentration and duration. Participants highlighted the importance of selecting a biologically relevant dose, tying the *in vitro* dose to the dose *in vivo* under the exposure scenario of interest. For example, the relevant dose metric for cytotoxicity is typically the inhaled dose per unit area. Potential dose metrics might include the external air concentration, the total inhaled deposited dose, or the inhaled dose deposited in a particular respiratory region, or amount absorbed (uptake) into cells (Phalen et al., 2021). Deposited dose is generally preferred over the external air concentration, as a better description of the amount of the chemical interacting with the target tissue. The particle size distribution (e.g.*,* mass median aerodynamic diameter [MMAD] and geometric standard deviation [GSD]) are key determinants of particle deposition, and these in turn depend on the form of the product (e.g.*,* spray bottle, pump, aerosol). The same cleaning product formulation in different types of packaging can produce different particle size distribution profiles. Dosimetry models, which consider the respiratory tract physiology, different breathing conditions, and particle size distribution, are useful to translate exposure to delivered dose metrics and thereby help connect the *in vitro* exposure method to the *in vivo* exposure of interest as addressed further in Section 6.2. Application of dosimetry models allows for determination of the dose delivered to various respiratory regions under the physiological breathing condition(s) of interest such as breathing mode (nasal, oronasal, or mouth breathing only) and breathing rate (Jarabek et al., 2005; Kuempel et al., 2015; Asgari et al., 2021). The user or bystander respiratory tract physiology and breathing rate should be considered in the *in vitro* cell/tissue test system (Section 4).

Participants noted that dosimetry models can calculate a wide variety of dose metrics, reflecting the tissue dose under the human *in vivo* exposure conditions, and informing the target delivered dose level for the *in vitro* respiratory irritation model. Deposition can be calculated on a regional basis, or models may make more localized predictions, helping to inform potential target cell types. Deposition can be calculated for a cleaning product formulation or for an individual ingredient. Depending on the physicochemical properties of the inhaled aerosol or gas, other potential dose metrics include flux to the tissue (a measure of mass per area per unit time) (e.g.*,* Kimbell et al. (2001)) or measures of the amount of reactivity within the tissue, such as DNA-protein crosslinks (e.g.*,* Conolly et al. (2023)). Some models also account for systemic absorption and distribution (e.g.*,* Sweeney et al. (2013)). In the case of a mixture, such as a cleaning product, the flux could potentially vary for each chemical although flux of the mixture is what would be needed as input to such models and could potentially be adjusted by molecular diffusivity for individual components. One participant noted that the flux of that chemical could be influenced by the mixture itself. Some participants opined that incorporation of flux into dosimetry models for respiratory irritation could add unnecessary complexity to the model, but others noted that it is a fundamental input parameter to compute internal dose of most models (e.g., PBPK, CFD, single pass mass transfer) and is easily characterized.

Some participants discussed the additional utility of dosimetry models (e.g., the MPPD model, the International Commission on Radiological Protection Human Respiratory Tract Model [ICRP HRTM], and computational fluid dynamics [CFD] or computational fluid particle dynamics [CFPD] models) in informing selection of the relevant *in vitro* cell/tissue test system by aiding in identification of the respiratory tract region or cell types with the greatest deposition or flux (ICRP, 2015; Corley et al., 2021; OECD, 2022; Ramanarayanan et al., 2022; ARA (Applied Research Associates), 2024). Alternatively, participants discussed that the most sensitive respiratory tract region or cell type, rather than the region with the greatest deposition, could be used as the basis for the *in vitro* cell/tissue test system. Based on the description of particle sizes generated from cleaning product use as >10 μm, it is assumed that it is unlikely for a significant portion of the aerosol distribution to reach the pulmonary region, but this depends on the geometric size distribution. Importantly, post-generation characteristics of aerosols should also be evaluated. For example, evaporation of volatile components can result in a reduction of aerodynamic diameter between generation, inhalation, and deposition. Consideration of the entire particle size distribution is also important, and if the distribution includes particles small enough to reach the pulmonary region, the implication of their exposure needs to be considered.

In selecting or designing an *in vitro* cell/tissue test system, one must consider the biological relevance of the cells or tissues exposed *in vitro* to those that are exposed *in vivo*, since different cell types may have qualitatively or quantitatively different responses to the same exposure (Faber et al., 2020). Considerations related to cell and tissue test system selection based on deposition distribution and anchoring to AOPs are discussed in further detail in the *In Vitro* Cell/Tissue Test Systems section (Section 4) of this report.

Participants noted that exhalation and clearance *in vivo* have an impact on the tissue dose, but these processes are not well characterized or represented in *vitro* assays. For particles, *in vivo* clearance mechanisms such as mucociliary clearance or removal via alveolar macrophages can reduce the tissue dose; particle transport and deposition during *in vivo* exhalation can also affect local cell/tissue dose. Some participants considered the lack of clearance mechanisms *in vitro* to be a challenge in utilizing dosimetry models to inform dose selection and duration of exposure. Other participants noted the utility of dosimetry models to predict the net deposition (*i.e.,* deposition occurring on both inhalation and exhalation) and that computational fluid-particle dynamics (CFPD) models can predict doses localized to specific cell types, to aid in comparing *in vivo* and *in vitro* doses. Some participants emphasized the use of washing and recovery periods *in vitro* to simulate *in vivo* clearance but acknowledged that this does not capture the intricacies of the biological process of clearance *in vivo*; further, it was noted that washing may remove important components, such as ELF. The ELF plays several complex roles that were acknowledged, but not fully explored at the workshop, including metabolic activation of chemicals (Pulfer et al., 2005; Squadrito et al., 2010; Pauluhn, 2021). *In vitro* cell/tissue test systems may be unable to capture the complex *in vivo* dynamics of ELF production and transport, or of tissue remodeling after chemical insult that may impact exposure *in vivo* (Mudway and Kelly, 2000; Ng et al., 2004; Henderson, 2005; Ciencewicki et al., 2008; Chanez and Bourdin, 2008; Darquenne, 2012; U.S. EPA, 2019b; US EPA, 2020). The major implication of the inability to adequately represent respiratory tract clearance *in vitro* is uncertainty regarding how accurately *in vitro* dose reflects *in vivo* exposure and effects. Some participants wondered whether the lack of such clearance mechanisms *in vitro* allows for use of shorter duration exposures to simulate the human *in vivo* exposures. Other participants noted that consideration of ADME and MOA is important. For example, the relevant dose metric may be the parent or the metabolite, and peak versus area under the curve (AUC) for either might be the most relevant. Generally, some participants recommended that better characterization of the *in vitro* dose is needed to build confidence in the use of *in vitro* models.

### 3.2 Technical considerations in *in vitro* exposure

In addition to tailoring the *in vitro* exposure design based on the pertinent *in vivo* exposure scenario(s), there are technical considerations regarding basics of study design and quality that are important in all scientific endeavors, *in vitro* and *in vivo* alike. Just as detailed and accurate documentation of exposure protocols are necessary for *in vivo* studies, similarly detailed documentation is needed for *in vitro* studies (Percie du Sert et al., 2020a; Percie du Sert et al., 2020b; Whalan et al., 2019; Wong, 2007). For example, documentation of the exposure system should include the accurate identification and characterization of the cleaning product and/or ingredient, exposures utilized and details regarding preparation of dilutions, exposure method, analytical methods, exposure duration, and washing protocols if used, among others. In addition, the rationale for the exposure protocol should be included. The participants addressed three categories of important technical considerations for the use of *in vitro* NAMs in evaluation of the respiratory irritation of cleaning products and ingredients: exposure delivery method, dose quantification, and utilization of controls.

#### 3.2.1 Exposure delivery method

**FIGURE 1:**
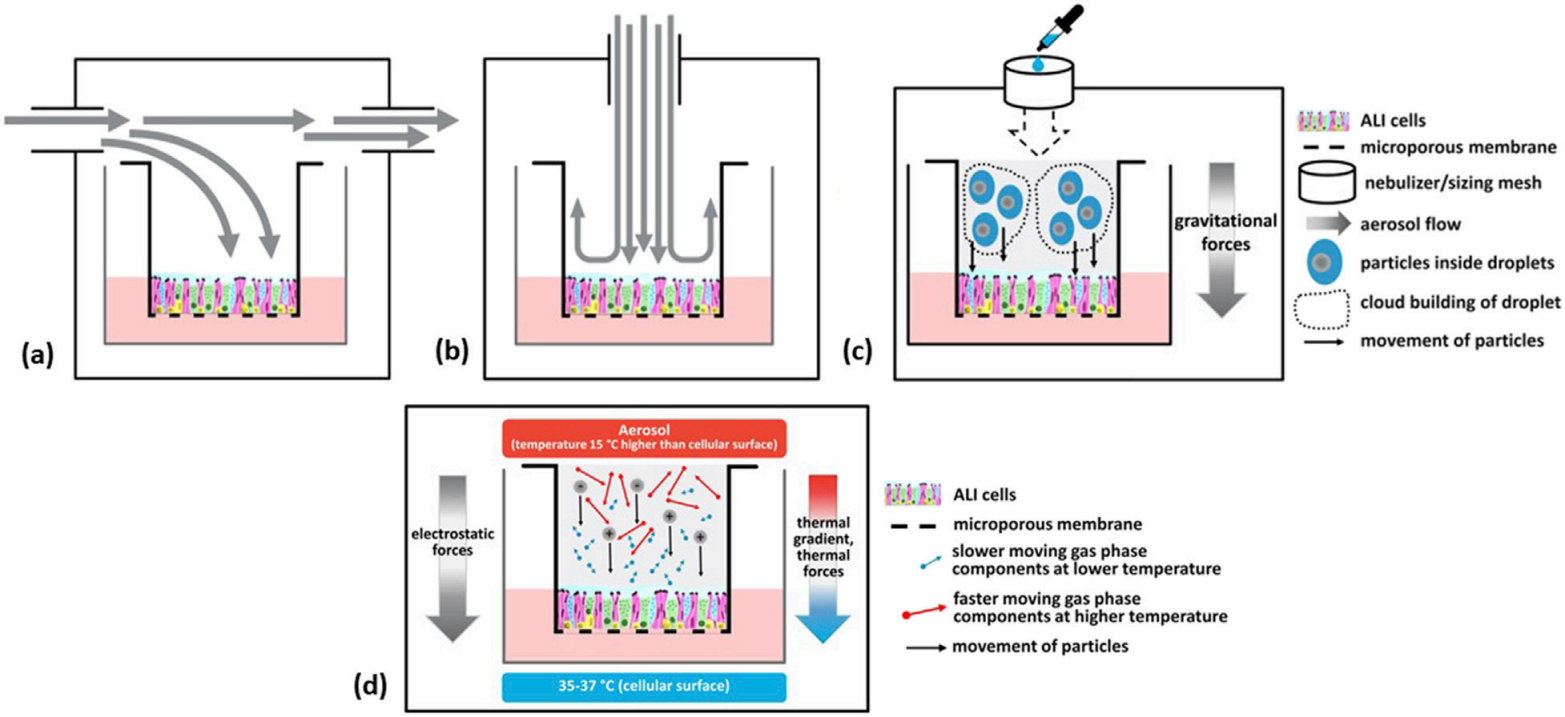
Schematics of various air-liquid interface (ALI) exposure systems. Continuous flow exposure systems include parallel horizontal **(A)** and perpendicular **(B)** that can provide for alignment of fluid dynamics; or cloud (droplet sedimentation) in an incubator/box type or stagnation point flow type that relies on gravitational forces **(C)**. Electrostatic forces or thermal gradients may be used to enhance deposition **(D)**. (Figure courtesy of A.M. Jarabek).

The three main types of exposure delivery systems that the participants discussed were: (1) submerged culture exposures (i.e., cultures that have never been cultures under ALI conditions), (2) direct liquid application to cells that have been grown at the ALI, and (3) ALI exposures. ALI exposure systems include continuous flow (parallel or incubator/box type and perpendicular or stagnation point flow type) that can provide for alignment of fluid dynamics, and cloud or droplet sedimentation types (e.g., ALICE-Cloud; Vitrocell^®^ Cloud) that rely on gravitational settling (Lewinski et al., 2017; Lacroix et al., 2018; Braakhuis et al., 2023). Various ALI exposure systems are illustrated schematically in Figure 1. Continuous flow systems are used for gases, complex mixtures, or particles of chemicals or materials which are available in larger quantities (several g) under a constant delivery for longer exposure durations. Cloud systems are used for single chemical droplet sedimentation or dry powders of materials that are scarce or expensive and for shorter exposure durations. The three main types of exposure delivery systems introduced at the beginning of this paragraph are discussed in greater detail in the following paragraphs.

It was acknowledged that these exposure methods coupled with cell/tissue systems represent different degrees of biological fidelity pertaining to the endpoints and nature of the responses. The level of biological fidelity required for the exposure depends on the exposure scenario being modeled. Numerous viewpoints regarding the appropriate use of these three exposure systems, as well as the degree of biological fidelity, were expressed by participants. Notably, discussions largely excluded detailed aspects of exposure relating to electrostatic or thermophoresis particle deposition, lung-on-a-chip technologies, and hPCLS.

Some participants felt that submerged culture exposures have limited or no value in respiratory irritation hazard and risk assessment due to a high degree of uncertainty and lack of biological fidelity (Lenz et al., 2009); others asserted that the ease of conducting submerged exposures make them useful for certain applications, such as high-throughput screening.

**FIGURE 2:**
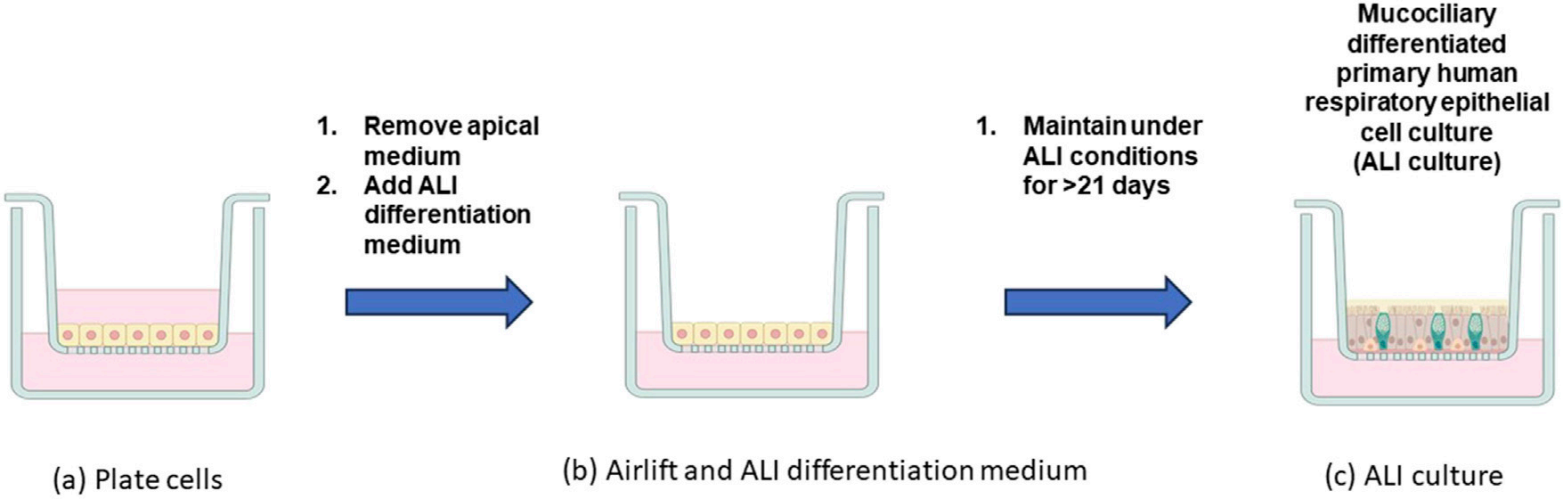
Differentiation of primary human respiratory epithelial cell models. Cells are plated on cell culture inserts containing porous membranes **(A)**. After the cells reach confluence, the apical medium is removed (“airlifted”), and the basolateral medium is replaced with ALI differentiation medium **(B)**. The airlifted cultures are maintained under ALI conditions for >21 days, resulting in the (differentiated) ALI culture **(C)**. Created with BioRender. (Figure courtesy of S. McCullough).

In contrast to submerged culture, ALI culture systems allow for the basal surface of the cells to be maintained on a membrane in contact with cell medium while the apical surface of the cells are exposed to air with the toxicant. Establishing and maintaining ALI conditions is critical for the *in vitro* differentiation of primary human respiratory epithelial cell cultures (Figure 2) (Fulcher et al., 2005; Ross et al., 2007; Rayner et al., 2019; Randell et al., 2011; Pezzulo et al., 2011; Kouthouridis et al., 2021). The generation of ALI respiratory tract models involves plating primary respiratory epithelial cells on cell culture inserts containing porous membranes (Figure 2A). Once they reach confluence, the apical medium is removed (“airlifted”) and the basolateral medium is replaced with ALI differentiation medium (Figure 2B). Airlifted cultures are then maintained under ALI conditions, with regular basolateral medium changes, for >21 days (actual duration differs based on the medium formulation used and how the completion of differentiation is defined). Following the ALI differentiation period, nasal and tracheobronchial cultures exhibit columnar ciliated epithelial cells with beating cilia, goblet cells that secrete mucus (shown in yellow), and basal epithelial cells (Figure 2C). A549 adenocarcinoma cells maintained under ALI conditions for extended periods of time (i.e., weeks) can differentiate to express type I and type II pneumocyte markers (Wu et al., 2017). As noted in Figure 1, ALI exposures utilize different delivery systems to expose cells at the ALI to gases, vapors, or aerosols. ALI exposure systems using various cell/tissue models represent promising exposure systems for nanoparticles, and they have been shown to provide transferability and reproducibility (Braakhuis et al., 2023). Recommendations for refinement of ALI exposure systems include developing a stepwise standard operating procedure (SOP) for operation and training personnel (Braakhuis et al., 2023).

**FIGURE 3:**
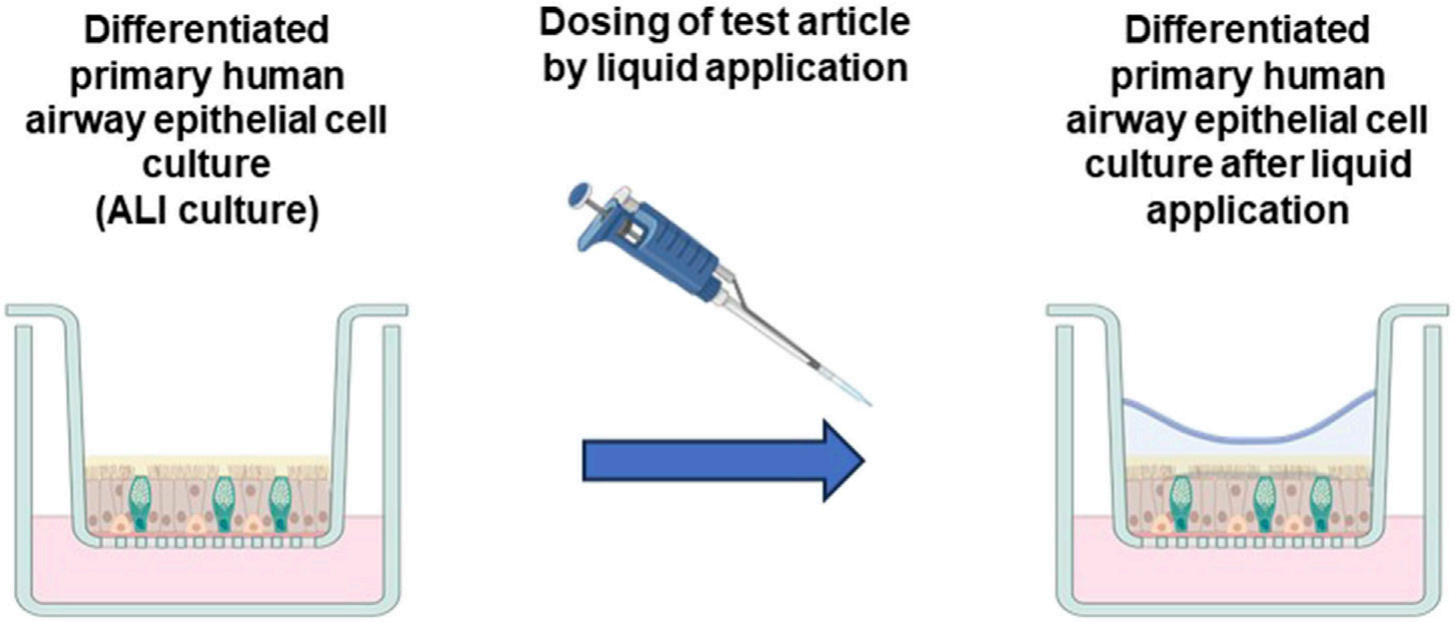
Transition of ALI cultures to liquid-liquid interface by liquid application dosing. ALI respiratory tract models are differentiated under ALI conditions, which involve exposure of the apical culture surface to ambient air and mimic the environment of the respiratory epithelium *in vivo*. Test article dosing by liquid application involves the addition of liquid to the apical culture surface, thus abolishing ALI conditions and resulting in a liquid-liquid interface. Created with BioRender. (Figure courtesy of S. McCullough).

Direct liquid application involves applying small volumes of test chemicals to the apical surface of ALI cultures; the delivery liquid may or may not be washed off at various durations, depending on the design of the experiment. Importantly, however, the addition of the liquid abolishes ALI conditions and results in a liquid-liquid interface (Figure 3). Reported usage of liquid application dosing includes a wide range of volumes; however, the ability of those volumes to completely cover the cell layer at the beginning and end of experimental exposures is typically not demonstrated. Further, the effect of applying the vehicle liquid on ALI cultures is similarly not demonstrated. A recently published study empirically determined the smallest liquid volume that would maintain complete coverage of ALI cultures for a 24-hour exposure and evaluated the effects of those conditions on ALI culture physiology (Mallek et al., 2024). This study demonstrated that application of liquid alone (i.e., in the absence of a test article) caused substantial changes to the physiology of ALI-differentiated primary human bronchial epithelial cells, including reduction in epithelial barrier integrity, activation of several cellular signaling pathways, and induction of pro-inflammatory cytokines and growth factors (Mallek et al., 2024). These changes were consistent with a range of respiratory diseases, as well as the effects of inhaled irritants, and the authors indicated that the effects of liquid application alone were likely to confound the interpretation of test article exposures. The observations reported in the Mallek et al. (2024) study were made with a single liquid vehicle at two liquid exposure durations. Thus, additional studies are required to determine whether similar effects occur under other liquid dosing conditions. The Mallek et al. (2024) study also demonstrated that commonly used applied liquid volumes, when applied to larger membrane diameters, do not maintain complete coverage of the cell layer after 24 h of exposure. While small volumes are typically used to minimize applied liquid depth, Mallek et al. asserted that the ability to maintain consistent coverage of the cell layer and thus avoid inconsistencies in culture conditions and test article exposures should be demonstrated for each set of exposure conditions used.

Some participants asserted that ALI exposures may be more biologically relevant than submerged exposures or direct liquid application to cells that were grown at the ALI for inhaled exposure to cleaning products and ingredients with intended applications as aerosols or sprays. Participants recognized that limitations of sedimentation cloud systems may include an inability to characterize how airflow affects particle transport and deposition *in vitro*, so that translation to *in vivo* particle sizes or airway concentrations is limited. Others felt that the value of data from an ALI exposure system outweighs the associated costs and challenges. Introduction of salts to the cleaning product for the nebulizer to create the exposure clouds is another limitation of sedimentation type ALI systems. In contrast to continuous flow systems, which allow for a slower deposition and accumulation of test article on exposed cultures, cloud systems deliver one or more bolus dose(s) of the test article. While cloud exposures can be highly relevant for the modeling of brief exposures (e.g., exposure of a consumer during the use of a cleaning product), they may not represent the kinetics of exposure that occur in longer occupational exposures (e.g., continuous exposure during an eight-hour workday). Importantly, the generation of large diameter liquid droplets for cloud exposures may result in the sedimentation of total liquid volumes over a short period of time that could be similar conditions to liquid application (Loret et al., 2016). When using cloud-based exposures, actual liquid volume deposition should be evaluated and comparisons between vehicle and incubator control cultures should be conducted to evaluate effects of the exposure method.

Some participants said that, in their experience, direct liquid application to cells that were grown at the ALI allows greater ease of conducting experiments and for more control of dosing than cloud exposure; but the range of ALI exposure systems is noted in the literature as providing a wide range of optimized fluid dynamics and effective cell contact (Lacroix et al., 2018). Finally, some participants mentioned some of the challenges of the ALI exposures, such as creating a setup that delivers a physiologically appropriate flow of material to the cell surface (Lacroix et al., 2018), but these considerations can be addressed with proper characterization (Lacroix et al., 2018; Braakhuis et al., 2023) or may be addressed by other emerging *in vitro* systems, such as lung-on-a-chip technologies (Bajaj et al., 2016).

The physicochemical properties of the cleaning product or ingredient may necessitate use of either liquid application or ALI exposures. For example, participants noted that ALI exposures are more appropriate for the delivery of volatile compounds that are insoluble in liquids (Zavala et al., 2018; Mistry et al., 2020). In another example of the importance of physicochemical properties, the INSPIRE project (*IN vitro* System to Predict Respiratory toxicity) required a modification to methods related to the mode of exposure, as vapor exposures had to be reduced in duration (from 1 h to 30 min) because the silanes being tested hydrolyzed rapidly in humid conditions (Sharma et al., 2023). This is likely a widespread issue for both volatile organic compounds (VOCs) and reactive gases given that ALI exposures require high relative humidity (RH) to mimic physiological conditions. Both RH and exposure concentration should be measured to address this concern, as nominal concentrations will likely be inaccurate and do not reflect actual exposure concentrations. Other participants noted that other, less-characterized, exposure methods may provide approaches for testing materials with low solubility in aqueous solutions, or that degrade in water-based solvents. An example of such methods includes the microvolume deposition of dimethyl sulfoxide (DMSO)-based liquids in the form of very small volume droplets (Behrsing et al., 2017). Participants recognized that some physicochemical properties, such as viscosity, may limit the feasibility for ALI exposures, necessitating liquid exposure. However, viscosity would decrease substantially when the ingredient is incorporated into the cleaning product formulation, which could then be tested via ALI exposure.

Participants also acknowledged that the exposure delivery method can impact the biological properties of the *in vitro* cell/tissue model and the toxicity of some xenobiotics. For example, direct liquid applications to an *in vitro* differentiated primary respiratory cell model altered the transcriptome, biological pathways, activation of several cellular signaling pathways, induced the secretion of pro-inflammatory cytokines and growth factors, and compromised epithelial barrier function relative to cultures maintained at the ALI and exposed to the same delivered dose (Mallek et al., 2024). Similarly, some participants postulated that powders of low or minimal toxicity may become toxic once mixed with the mucus (irrespective of dosing method), due to facilitated delivery into cells by the liquid components of mucus and/or formulation. Another example was that exposure to zinc oxide nanoparticles resulted in different toxic responses following submerged liquid and ALI exposures (Lenz et al., 2009). These studies demonstrate that (1) adherence to recommendations of the exposure system manufacturer for operating parameters is important; (2) biological responses may differ based on the exposure method used rather than the chemical, (3) biological changes should be interpreted in the context of the exposure application to identify chemically induced toxicological effects, and (4) biologically relevant systems (e.g., delivery method, particle size, chemistry, etc.) should be utilized to predict *in vivo* responses.

#### 3.2.2 Measurement and quantification

The administered concentration, the dose delivered to the *in vitro* system, and the intracellular dose represent three different important aspects of exposure. It was noted that the internal dose is the driver of the toxicological response and is the most appropriate dose metric, although it is difficult to measure and is a product of ADME. Therefore, the delivered dose, rather than the administered concentration, should be measured (Schmid and Cassee, 2017; Phalen et al., 2021). Methods for measuring the dose delivered, but not taken up by the cells, were discussed by the participants. The choice of method for measuring the concentration delivered to the cells depends on the physicochemical properties of the cleaning product.

The participants noted that one measuring approach used filter disks to collect particles to estimate deposition efficiency and determine deposition uniformity across cell culture inserts (Oldham et al., 2020b). Different deposition efficiencies based on particle size were demonstrated (Oldham et al., 2020b). Others noted that limitations of the filter paper approach include low deposition efficiency and that deposition on a dry filter disk does not mimic deposition on a moist cell surface.

In contrast, an integrated quartz crystal microbalance (QCM) was used in an inter-laboratory effort to harmonize delivered dose from the Vitrocell^®^ Cloud system (Bannuscher et al., 2022). Use of the QCM addressed variability observed by measurement of deposition fraction on transwell inserts alone (Ding et al., 2020; Bannuscher et al., 2022). Measurements from the QCM can be used to verify homogeneity of the exposure in the chamber and across transwell inserts, which may not always occur; however, quantifying exposure homogeneity may be limited by the ability to use the QCM in wells in the middle rows/columns of devices intended for use with 12- or 24-well inserts. Regardless of the measurement method, adherence to recommended or standardized procedures or SOPs helps to promote homogeneity and reproducibility in the net deposition (Ding et al., 2020; Bannuscher et al., 2022).

Participants also acknowledged the influence of flow in the chamber on exposure delivery, especially when comparing between parallel and perpendicular flow delivery systems. Uniform mixing of aerosols was obtained with use of a dilution unit (Kuczaj et al., 2016), but others reported heterogeneous mixing of aerosols in flow chambers in a perpendicular flow system (Oldham et al., 2020a; Oldham et al., 2020b).

Participants also noted that, similar to challenges with *in vivo* chamber exposures, the measurement of a concentration in the chamber does not represent the dose delivered to the cells. Accurately characterizing the dose to the cells in ALI systems is challenging because of the variability in particle deposition in ALI systems, which is influenced by the physicochemical properties of the aerosols and the type of exposure system utilized by the study (Oldham et al., 2020a; Oldham et al., 2020b; Steiner et al., 2017). For example, in continuous flow aerosol exposure systems, losses of aerosol constituents at the inner surfaces of the exposure system components (e.g., tubing, trumpets, etc.) are commonly observed due to adsorption and other particle transport mechanisms such as impaction, and sedimentation (Steiner et al., 2018; Wong, 2007; Yi et al., 2013). In sedimentation aerosol exposure systems (*e.g.,* Vitrocell^®^ Cloud), the aerosol droplets tend to adhere to the inner surfaces of the chamber (polycarbonate) and the nebulizer (Bannuscher et al., 2022). The aerosolization and deposition efficiency vary for different materials based on their physicochemical properties, such as hydrophilicity. While some of the materials may react with the polycarbonate parts of the chamber and nebulizer (with high concentrations and sufficient contact time), participants noted that this reaction may not have a significant effect on particle deposition due to comparatively shorter exposure duration (i.e., 5–10 min). Characterizing the settling velocity and time for particles generated by nebulizers using varying mesh sizes for the cloud delivery system will be important for quantifying dose delivered to the cells. Hydrophobic constituents may also adhere to the materials of cell culture insert (e.g., polyester terephthalate [PET]) at the ALI (Steiner et al., 2018). Additional parameters that affect deposition of the aerosol at the ALI include solubility in mucus, culture medium, volume, and transwell surface area, and volume (Steiner et al., 2018).

For volatile chemicals, a known volume of trapping liquid can be placed within the exposure chamber to collect deposited material. The total amount of material deposited can be determined based on the concentration of the material in the known volume of trapping liquid. Steiner et al. (2018) demonstrated that the trapping liquid influences the delivery efficiencies of constituents in smoke exposures.

For liquid application exposures, representative samples for analysis can be taken at dosing to confirm the concentration, homogeneity, and the actual (rather than nominal) exposure applied in the test system. The homogeneity has been demonstrated to be dependent on both the volume applied as well as the duration of the application (Mallek et al., 2024). Participants noted that this application concentration does not capture the dose that the cells are exposed to or take up, but rather the applied exposure dose. Further, participants recognized that, as for aerosol exposures, hydrophobic constituents in submerged cultures may also adhere to the materials of the cell culture insert (e.g., PET), and that the solubility in mucus, surface area, and volume may also affect deposition (Steiner et al., 2018).

In addition to the measurement of the exposure of the cleaning product or ingredient, participants noted that other experimental conditions should be recorded due to their potential to influence both the amount of test article delivered and the results of the *in vitro* assays. Such factors include the temperature, humidity, particle size distribution (e.g*.,* MMAD, GSD), and density. Temperature, humidity, and ventilation can affect the experimental conditions and may or may not be controlled; they should be reported to aid in interpretation of results. Some participants felt that these challenges are not unique to *in vitro* methods and present a challenge in *vivo* exposures as well, while others noted the use of controlled temperature and humidity *in vivo*. For liquid exposures, some participants noted that a unique challenge remains the influence of the volume of test chemical on the assay. For example, when determining the threshold for irritation for a cleaning product mixture, different volumes may be required to achieve different exposure doses, as dilution changes the composition of the mixture. This may impact the dose that directly interacts with the apical surface of the cells. Other participants noted that many assays require the use of a specific volume, and therefore this issue is not always pertinent.

#### 3.2.3 Utilization of exposure controls

Use of appropriate controls for the *in vitro* test method selected is essential to have confidence in the data and allow for proper interpretation in the context of the control responses. Six broad types of controls were identified for use *in vitro* respiratory irritation assays: (1) vehicle controls, (2) cleaning formulation solvent controls, (3) single blank (processing) controls, (4) double blank (no processing and no dose) controls, (5) negative controls, and (6) positive controls. However, it was acknowledged that additional controls may be needed for certain assays and endpoints. Vehicle controls are essential to evaluate whether the vehicle used in the assay, rather than the cleaning product or ingredient being tested, is causing the observed outcome in the assay. If the product is intended to be diluted prior to use, the diluent is also tested as a vehicle control. Selection of the vehicle control is impacted by the assay endpoint of interest. In cases where more than one vehicle control is used, all vehicles should be characterized in the assay to evaluate the variability elicited by choice of vehicle. Solvent controls may be necessary to evaluate the effect of the cleaning product formulation solvent on the outcome of the assay. For example, if the cleaning product solvent is ethanol, the toxicity of ethanol should be evaluated separately, in addition to the toxicity of the entire cleaning product formulation. In some cases, the cleaning product solvent may inform the choice of the assay vehicle such that both are the same. Therefore, the solvent control may be the same as the assay vehicle control (e.g.*,* both a water solvent and a water vehicle) or different (e.g.*,* ethanol solvent control and a water vehicle control). Single blank or processing controls, which are not dosed but undergo processing, may also be necessary to evaluate the system itself. For example, in an ALI exposure chamber, while a vehicle control may be aerosolized water droplets, the blank controls may consist of pumped air or no air current (i.e., an incubator control). Double blank controls are those that receive no dosing and do not undergo the processing. For example, when using a wash, the double blank control will not be washed, to evaluate the effect of that process on the outcome of the assay. The use of positive and negative controls demonstrates that the *in vitro* test system is producing the expected response (i.e., known respiratory non-irritants are eliciting negative results and known respiratory irritants are eliciting positive results). Identification of reference chemicals to serve as positive controls that elicit positive responses in the selected *in vitro* cell/tissue system is critical to avoid false negative results (Bisig et al., 2019). Further, the use of controls contributes to characterizing the nature of the response (e.g.*,* benchmark level) as well as intra- and interlaboratory variability in the assays (OECD, 2018a).

### 3.3 Conclusion

Substantial advancements have been made in recent years in the exposure methods used for assessment of respiratory toxicity. The choice of the appropriate exposure design for an *in vitro* respiratory irritation assay depends on consideration of the likely effect and the intended use of the data based on the purpose and assessment type. Thus, there is not a single correct way to design the exposure across *in vitro* respiratory irritation assessments, but there are considerations that should be kept in mind when choosing a particular exposure system. One such major consideration is the physicochemical properties of the cleaning products and ingredients; a comprehensive review of these properties and their impact on selection of exposure methods would be useful guidance for developers and users of NAM technologies targeting these chemicals. The exposure scenario has implications in the *in vitro* cell/tissue test system selection (e.g.*,* relevant cells or tissues based on dosimetry) and for the risk assessment (e.g.*,* reducing uncertainties). The exposure methods utilized in an *in vitro* cell/tissue test system of respiratory irritation, and the appropriate justification and documentation of these methods, guide and can limit the scope of interpretation of the resulting data. This will help to make the resulting data useful for the desired application in screening or risk assessment.

## 4 *In Vitro* cell/tissue test systems

### 4.1 Considerations in characterization and standardization of *in vitro* cell/tissue test systems, assays, and reporting

To maximize the use of *in vitro* NAMs to predict respiratory irritation in response to cleaning products, the participants identified a need to use well-characterized systems and standardized procedures and develop reporting standards. These tools will facilitate reproducibility across laboratories and interpretation of data generated using *in vitro* methods. This section (Section 4) outlines some considerations relevant to these systems, procedures, and reporting standards, but is not meant to cover all possible items.

Variability in *in vitro* methods and assays can be due to several factors such as the type and “lifespan” of cell/tissue test system, culturing conditions (medium components, cell seeding density, etc.), and how the assay is performed and reported. These variations can affect the outcomes of assays, and thus pose a challenge to the use, integration, and interpretation of the results. Some participants noted that *in vivo* studies face similar challenges, with variation by species, strain, food and water, housing, and other factors. Consistent information from standardized reporting of assays can assist with the characterization of assays within defined study plans used to conduct those studies. This will, in turn, provide a clearer understanding of how differences in methods may contribute to variation in results.

Participants particularly noted an urgent need to be proactive in characterizing and potentially standardizing *in vitro* respiratory irritation assays and reporting, instead of merely summarizing comparisons or conducting retrospective analyses after data are gathered. Such characterization and standardization will facilitate the use of data from assays for a broader range of applications, including filling data gaps. As mentioned in Section 3, to further characterize assays and establish confidence in NAMs, *in vitro* test systems and assays also need to be considered in the context of Good Laboratory Practices (GLP). The principles outlined in the Guidance Document on Good *In Vitro* Method Practices (GIVIMP) (OECD, 2018a) and RIVER working group (2023) should also be considered, though GIVIMP does not have the same regulatory status as GLP and does not guarantee discussion of the rationale for choice of assay or test system. Critically, assays used in conjunction with *in vitro* respiratory tract models would benefit from improved reporting to aid in characterization.

The longer that any research proceeds without well-characterized and/or standardized assays, the more data that may need to be regenerated to fit into standardized assays and any necessary framework. In a broader context, Jarabek and Hines (2019) discussed a workflow for coherent integration of *in vitro* and other evidence across a range of risk assessment and regulatory applications, including sufficiency of metadata, transparency of assumptions, and explanation of applicability domain for assays. A recent paper on liver-chip methods provides one possible model approach for standardization of *in vitro* methods, including considerations of both technical and cost aspects (Ewart et al., 2022). One example of an effort towards standardization of inhalation toxicity testing (though not specific to respiratory irritation) is the Respiratory Toxicity (RespTox) Collaborative—an international, cross-sector consortium of experts conducting *in vitro* inhalation toxicity testing (publications in preparation). The collaborative was established for developing and gaining consensus on the minimum information reporting needs for different assays. Participants were unaware of any such efforts specific to *in vitro* respiratory irritation.

Conducting a literature survey or review would identify existing data and knowledge gaps to guide generation of additional information. For example, participants noted that identifying the data available regarding the appropriateness of different assays and models for different physicochemical properties of cleaning product ingredients or formulations is a key step towards assay standardization. There will be significant challenges for conducting this survey/review in the context of respiratory irritation and cleaning products or ingredients, and for any characterization and standardization effort more broadly. Publication bias will pose a challenge as negative results from assays are critical for overall understanding of the assays. Further, small differences in methodology can also produce different results, and so incomplete documentation of methods will also be a barrier to fully understanding assays and test systems. Therefore, engagement of stakeholders and those with hands-on knowledge is critical to ensure that this additional information is captured in the analysis, as these groups will likely have the best insight into unpublished results or missing methodological details. Workshops such as the one that is the basis for the current publication can play an important role in gathering perspectives from a diversity of scientists and can inform or supplement a literature survey/review. The following paragraphs outline some considerations that may be addressed in more detail by such a literature survey/review and/or future workshop, but this list is not exhaustive.

There is a wide range of available cell/tissue models that have been utilized to evaluate respiratory irritation potential *in vitro*. Most discussion at the workshop focused on primary monocultures versus cocultures, but the considerations apply more broadly to cell/tissue test system selection. First, resemblance between *in vivo* and *in vitro* responses needs to be considered when characterizing *in vitro* cell/tissue culture methods and developing standardized approaches. For example, primary cells and immortalized cell lines will respond differently under the same exposure conditions for some, but not all, assays and exposures. Therefore, understanding the differences in response and identifying which more closely resembles the *in vivo* response is critical in selection of the cell/tissue test system. Second, accessory cell types (e.g.*,* fibroblasts, macrophages) may act as mediators or moderators or exert more direct effects on the responses to exposure, even if these cells are not directly in contact with the chemical. A mechanistic understanding of respiratory irritation can provide information on when accessory cell types need to be included and which cell types should be considered. Third, the appropriateness of cell/tissue test systems is dependent on the KEs under investigation, which are affected by the physicochemical properties of the test article. For example, there are at least 41 cell types in the lung, including bronchiolar and alveolar epithelial cells, and different cell types are known to respond differently to the same compounds, due to their different biology (Parent, 2015; Harkema et al., 2018). Finally, it will be necessary to characterize the background response variability of the available *in vitro* cell/tissue test systems to differentiate test system variability from exposure-related effects (van der Zalm et al., 2022). Generally, the factors listed here inform test system selection, but both the feasibility of use and the access to relevant cell/tissue test system must be considered. For example, primary cells can be expected to be more representative of *in vivo* responses but there may be logistical or cost barriers to using them.

There is a tradeoff between phenotypic complexity of the *in vitro* cell/tissue test system and throughput. More complex systems (e.g.*,* ones including multiple cell types and/or 3D structure) tend to be more expensive and time-intensive to run, while the higher throughput systems tend to be less sophisticated but may not produce fully tissue-relevant data. These opposing forces will need to be balanced, and this balance may be prioritized differently depending on the purpose and stage of the assessment (Table 1).

Participants noted that some *in vitro* cell/tissue test systems or assays relevant to respiratory irritation will be easier to standardize than others. For example, ciliary beat frequency (CBF) can indicate impaired ciliary motility and mucociliary transport via mucin hypersecretion triggered by respiratory irritants, as seen in diseases such as asthma and cystic fibrosis (Bustamante-Marin and Ostrowski, 2017). CBF may be relatively easy to standardize and technical considerations and recommendations have been made (Behrsing et al., 2022). For technical proficiency in this endpoint, it will be key to record video with maximum contrast between light and dark, making it easier to identify the effect with appropriate analysis software. Additional examples were not discussed as part of this workshop, but similar suggestions and considerations for characterizing and standardizing assays would apply to other endpoints.

The medium used in a particular cell/tissue test system may affect what endpoints can be tested. Different medium choices can have an impact on physicochemical properties of the test article and have different effects on cell morphology/physiology/function and response in the same cell system (Saint-Criq et al., 2020; Leung et al., 2020; Lee et al., 2020). Each *in vitro* cell/tissue test system has particular medium requirements, especially for co-cultures with different media and media mixtures being used at various stages of culturing (Klein et al., 2013), and laboratories may use in-house formulations rather than commercial media. These in-house media are likely to differ across laboratories and therefore should be reported. Some commercially available test systems (e.g.*,* from Mattek and Epithelix) can be purchased with medium specifically developed for the test system. When new endpoint assays are implemented, they should be optimized to the manufacturer’s media whenever possible. Where the medium has to be changed, careful evaluation of the medium change should be conducted with suitable control groups.

Another consideration is that the choice of *in vitro* cell/tissue test systems, respiratory irritation assays, and modes of exposure depends on the physicochemical properties of test chemicals. An understanding of the relationship between different sets of physicochemical properties and the appropriate choice among emerging cell/tissue test systems will be useful. For example, the choice of hPCLS culture method has implications for hPCLS assays used for cleaning products with aerosol exposure. Aerosol exposures are possible using the hPCLS ALI (tissue culture insert) method, but not possible using hPCLS roller culture method or submerged cultures (Patel et al., 2021). More generally, in exposure at the ALI (e.g.*,* complex coculture or hPCLS), the test article (including solid, liquid, or aerosol) can be applied indirectly (by aerosolization) or directly (hand pipetted, automatically dispensed in a pattern distributed across the ALI surface (Behrsing et al., 2017)) onto the *in vitro* test system and thus is not required to be soluble in the chosen cell culture medium. In contrast, in submerged exposure of an *in vitro* monoculture (i.e., single layer, 2D cell culture), the test article needs to dissolve in the chosen cell culture medium, limiting the applicability of submerged culture systems. Mode of exposure can also have implications for interpretation of assay results. In one study, submerged exposures led to higher production of reactive oxygen species and secretion of IL-8 relative to cloud ALI exposures (Klein et al., 2013). Considerations of volatility of the test article also has implications for *in vitro* test systems and exposure. For example, volatile compounds may evaporate rapidly if dissolved in warm media. Different particle size distributions impact cellular uptake. Thus, the choice of *in vitro* assays, test systems, and modes of exposure is critical for any assessment of respiratory irritation.

In addition, the physicochemical properties of cleaning product formulations may differ from those of their individual chemical components, with associated implications for the assays that can be conducted. Testing a single chemical or component of a cleaning product alone, even at a dilution similar to that used in the final formulation, may not accurately reflect physicochemical properties and behavior of the ingredient in the final formulation.

### 4.2 Capturing population variability *in vitro*

It is important to characterize population variability in the context of a risk assessment model. This might be done using default approaches or using information on variability derived from *in vitro* assays. Susceptible populations are currently accounted for *post-hoc* by using uncertainty factors (UF) to address human variability, but *in vitro* cell/culture test systems may be able to provide or inform a quantitative understanding of variability, which can result in more scientifically informed UFs. However, for some risk assessment purposes (e.g.*,* screening), default UFs may be sufficient. UFs are discussed further in Section 6.4.

Possible sources of population variability include sex, age, race, geographical source, disease status, and susceptibility windows. Ambient air quality differs across geographical areas (Brody et al., 2002; Vrijheid, 2014), potentially resulting in different baseline characteristics in primary samples collected from donors in different areas. Susceptibility windows of interest noted were early development (Goldizen et al., 2016) and menopause (Ziller et al., 2023). Younger populations are often considered to be more at risk due to differences in dosimetry and respiratory tract development (Ginsberg et al., 2008; Ginsberg et al., 2017; Dietert et al., 2000). Participants also noted that some subpopulations may be *more* resilient (less susceptible) to respiratory irritation, though specific examples were not given.

Population variability might be dependent on the endpoint of interest, the chemical/formulation of interest, or both; these dependencies may influence the choice of *in vitro* cell/tissue culture model. As an example of the former, males and females are likely to have similar CBF of around 12–15 Hz in healthy adults (Roth et al., 1991; Tilley et al., 2015), but may have differences in airway inflammation and remodeling (Ekpruke and Silveyra, 2022).

One aspect of population variability and susceptible populations that is particularly relevant for the testing of cleaning products is consideration of the anticipated biological features (e.g.*,* ventilation rate and airway anatomy) of both the intended end-user and of those who may be unintentionally exposed in the presence of the end-user. *In vitro* cell/tissue test systems representative of the biology of some susceptible populations are available (Faber and McCullough, 2018). ALI-differentiated primary human bronchial epithelial cell models have been shown to exhibit inter-individual variability across donors (McCullough et al., 2016; Bowers et al., 2018; Bowers et al., 2021). The use of cryopreserved lung slices allows for consideration of population variability to some degree, at the cost of a greater level of complexity and study scope (Patel et al., 2023). Depending on the assessment purpose, it may be possible to address such considerations through selection of particular *in vitro* test systems.

## 5 Tiered testing framework/approach

The *in vitro* cell/tissue test systems breakout group proposed an option (described in Section 5.1) for development of an approach for *in vitro* test systems and assays to evaluate respiratory irritation for cleaning products, with room for customization to specific needs of each assessment. Some additional background and context relevant to the development and use of an approach are discussed in Section 5.2. This approach includes an initial tier of screening chemicals based on physicochemical characterization and computational approaches (Tier 1), followed by up to two tiers (Tiers 2 and 3) of testing in appropriate *in vitro* cell/tissue test systems informed by mechanistic understanding. This is explicitly not a recommended approach but is meant as an illustrative example of how such an approach might look. The participants acknowledge that this is a starting point and may need to be modified based on the outstanding questions (below), findings from a literature survey/review (above), and/or the specific problem formulation or goal of testing (Section 1). Depending on the purpose to which the approach is applied, some tiers may not be needed. Lower tiers (Tier 1 and possibly Tier 2) may be adequate for screening, while more sophisticated applications, including regulatory applications, may require elucidation of mechanisms or filling data gaps in higher tiers (Tier 2 and Tier 3) potentially coupled with data from *in vivo* cell/tissue test systems.

### 5.1 An illustrative option for a tiered approach to screen thousands of ingredients/mixtures

One option for a proposed tiered approach is presented herein. As noted above, this is explicitly not a recommendation but is meant as an illustrative example of how such an approach might be designed. Similar approaches have been proposed for evaluating natural complex substances for a range of endpoints, including local respiratory toxicity (Api et al., 2022; Clippinger et al., 2018a). Though not specific to respiratory irritation, this tiered approach for complex mixtures may also inform considerations relevant to cleaning product formulations.

#### 5.1.1 Tier 1: Physicochemical characterization and computational screening

The main goal of this tier would be to inform later testing. The goal at this level is explicitly not to identify test products or ingredients as non-irritants, but to identify appropriate assays and *in vitro* cell/tissue models that can be used to assess them. In addition, this tier could be used to “screen out” test articles that are not good candidates for use in cleaning products because they are highly likely to be irritants, though specific examples of physicochemical properties that would indicate that a test article is likely to be an irritant were not discussed in detail. Since cleaning product ingredient manufacturers and cleaning product formulators aim to use non-irritant ingredients/formulations, it does not make sense for ingredients/formulations that are identified in this tier as highly likely to be irritants to proceed to further testing. Screening out very likely irritants at this early stage could optimize the use of resources in the *in vitro* testing in Tiers 2 and 3. Ingredients/formulations that are identified in this tier as likely to be non-irritants, or those which may or may not be irritants, should proceed to further tiers, so that predictions can be confirmed or refuted with experimental data. The exact threshold to determine when an ingredient/formulation is “highly likely to be an irritant” was beyond the scope of this workshop, but may vary depending on the purpose of the assessment. Care may need to be taken to not be overly restrictive at this early stage, given the “screening level” nature of this tier.

Before performing any laboratory testing, computational approaches based on physicochemical properties and/or structural flags can identify appropriate test systems and exposure systems, and flag ingredients/formulations of obvious concern for respiratory irritation. Physicochemical properties should be known before beginning laboratory testing and need to be considered throughout the process. Consideration of physicochemical properties can guide users to choose *in vitro* cell/tissue test systems appropriate for such properties and can have a big impact on readout of tests. Similar to considerations for exposure systems (Section 3.3.1), some test systems or assays will be better for, or more sensitive to, certain chemistries.

#### 5.1.2 Tier 2: Simpler *in vitro* models

Due to considerations of cost, time, and throughput, *in vitro* testing of ingredients/formulations may begin with using a simpler cell/tissue test system (e.g., a simple 2D mono-culture using submerged exposure), with the aim of collecting data on multiple relevant endpoints from a single cell/tissue system (i.e., multiplex). The specific choice of cell/tissue test system in this tier may depend on the purpose of the assessment, and some purposes may demand a higher level of complexity than others. For example, if specific effector cells are expected to be involved in the irritation response, a co-culture test system including those cells would be preferred. The mode of exposure should also be considered at this stage. Submerged and liquid application exposures are faster, easier, and less expensive than ALI exposure, and so may be favored at this early step when possible. However, there are efforts to improve liquid application exposures (e.g., Behrsing et al. (2017), Mistry et al. (2020)), and this may enable the use of these systems for rapid screening.

To determine an ideal *in vitro* cell/tissue test system around which to base a “multiplex-centered” testing strategy, it is necessary to first identify the *in vitro* cell/tissue test system that addresses the greatest number of relevant KEs, then fill in gaps with other models. KEs can be selected from any established AOP or informed by a broader IATA that contributes additional evidence. This is especially relevant to respiratory irritation, given the lack of a well-developed and accepted AOP for this endpoint (Section 2). For example, inflammation may contribute to both irritation and sensitization (though it is not required for irritation). If there are relevant mechanistic steps that are not included in an AOP or IATA (or if an AOP or IATA do not yet exist), these steps should also be considered.

Multiplexing may be achieved by conducting non-destructive assays (i.e., monitoring various functional readouts that do not damage the structure or integrity of the cells) in an *in vitro* cell/tissue test system first, and then letting those findings guide the next steps in the process (which may include destructive testing). Cortesi and Giordano (2022) recently reviewed such assays for 3D cultures. For example, one could evaluate changes in CBF initially (a relatively non-destructive test) and then immediately assay mitochondrial toxicity (a destructive test such as the MTT [3-[4,5-dimethylthiazol-2-yl]-2,5 diphenyl tetrazolium bromide] assay). In this example, the value of assessing multiple endpoints in a single test system is prioritized over the challenge of conducting the more complex/expensive endpoint first; depending on the purpose, the relative priorities of these aspects may be different. Multiplexing may also be achieved by choosing a non-destructive test (such as WST-8 [2-(2-methoxy-4-nitrophenyl)-3-(4-nitrophenyl)-5-(2,4-disulfophenyl)-2*H*-tetrazolium, monosodium salt]) instead of a destructive test (such as MTT). This substitution would allow samples already used for WST-8 to be multiplexed for omics (Phillips et al., 2021) or other biochemical evaluation, or to be fixed for histological examination following assays for cell viability.

Results from submerged exposures in simple 2D monoculture (or co-cultures with accessory cells, if required) in this tier can determine whether progression to the next tier, with more complex cell/tissue test systems and/or modes of exposure, is warranted and can inform the selection of cell/tissue test systems and modes of exposure. Results from this tier can also increase the efficiency of testing in the following tier. For example, cleaning product manufacturers may be able to screen out ingredients/formulations that are clearly irritants in this earlier tier, thus reserving further testing for those that are unlikely to be irritants, or those that may or may not be irritants. For some assessment purposes (e.g., a simple hazard characterization), the results at this tier may not require progression to the next tier. For other purposes (e.g., a full regulatory risk assessment), the next tier (and more) would likely be required.

#### 5.1.3 Tier 3: More complex *in vitro* models

Based on outcomes from Tier 2, the aim in Tier 3 is to address as many relevant KEs or other mechanistic supporting endpoints as possible in a single test cell/tissue system (i.e., maximize data output from a single experimental model), and use additional test systems to address additional KEs and/or other mechanistic endpoints not addressed by the primary test system. An example from respiratory sensitization may provide a model for multiplexing endpoints in a single test system (Gibb and Sayes, 2022). Such multiplexing may be a challenge for some endpoints, considering that other, well-characterized endpoints (e.g., skin sensitization) require multiple assays that are not amenable to multiplexing to address separate KEs. However, this challenge may not be relevant for respiratory irritation. Table 2 presents an illustrative example of how two different 3D culture systems (RHuA and hPCLS) can each cover a range of endpoints relevant to respiratory irritation (as currently conducted at one organization at the time of publication; some specific model-endpoint capabilities may change as additional assay capabilities are developed or may be different between organizations). For example, 3D RHuA can be used for tight junction integrity, mucociliary clearance, goblet cell increase, macrophage activation, and mucin expression, while 3D hPCLS can be used for macrophage activation, extracellular matrix (ECM) deposition, and airway contractility. Depending on the particular purpose and the results from the simpler Tier 2 testing, one or both 3D culture systems could be chosen. Choice of test systems will also depend on the capabilities of the performing laboratory. Participants also mentioned that specific pairing of assays and irritation KEs would be beneficial, but specifics were not discussed at the workshop. Future work could build on Table 2 to include more specifics on which assays can address specific KEs to contribute data to a framework. Progress toward an AOP would be complementary to this future work.

**TABLE 2:** Example of Endpoints that can be Tested in Different Cell/Tissue Test Systems.

	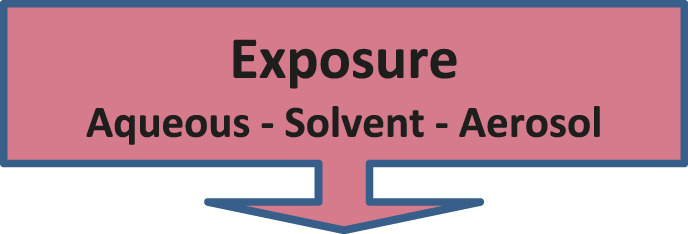 Test system	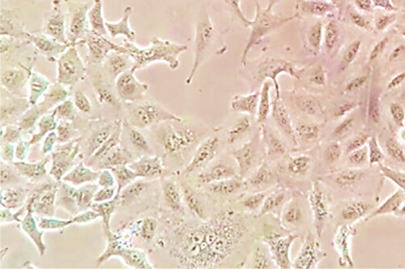	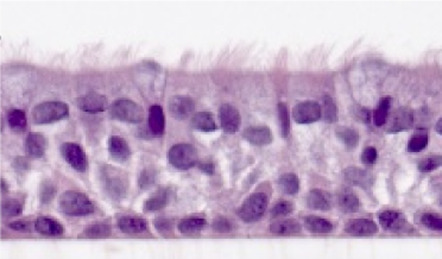	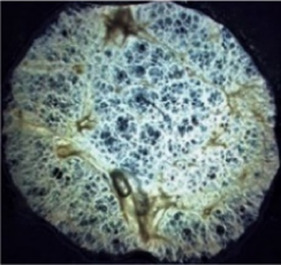
	Biomarkers/Events	2D cells	3D RHuA	3D hPCLS
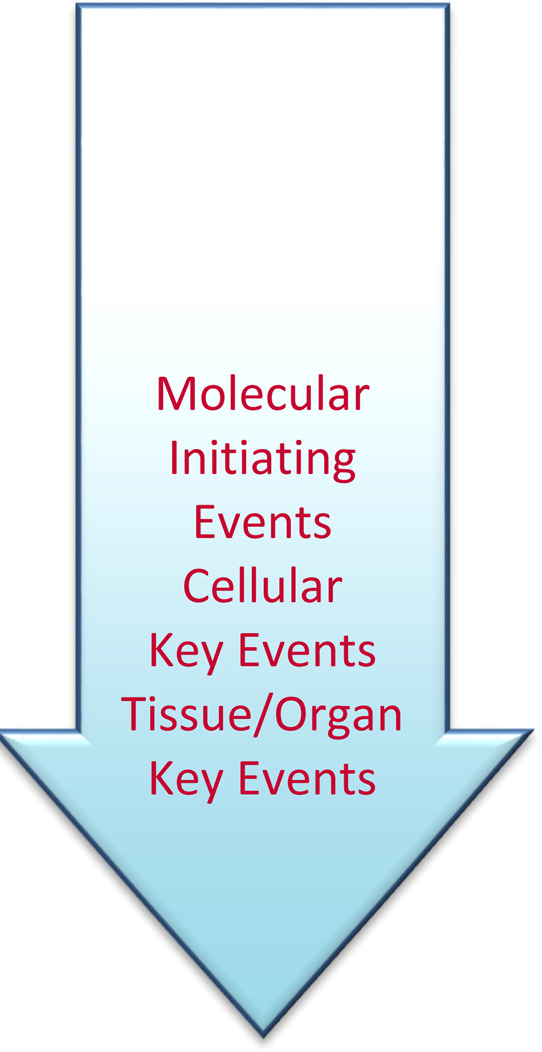	Oxidative Stress	✓	✓	✓
	DNA Binding/Strand Breaks	✓	✓	✓
	Mitotoxicity	✓	✓	✓
	Cytotoxicity	✓	✓	✓
	Viability	✓	✓	✓
	Macrophage Activation^a^		✓	✓
	Cytokine/Chemokine Response	✓	✓	✓
	Tight Junction Integrity		☑	
	ECM Deposition		✓	✓
	ECM stiffness	✓	✓	☑
	Spatial context (histology)^b^		✓	✓
	Mucociliary Clearance^c^		✓	✓
	Airway Contractility			☑
	Vessel contractility			☑
	Goblet Cell Increase		☑	
	Mucin/surfactant Expression		✓	✓
	*In vivo*-comparable tissue dosimetry^4^		☑	

These examples reflect capabilities at the time of the workshop discussion/publication and may change over time or differ across institutions. 3D RHuA = 3D reconstructed human airway. 3D hPCLS, = 3D human precision cut lung slices. (ECM, extracellular matrix). A checkmark (✓) indicates that a given test system can address a biomarker/event. A checked box (☑) indicates that the biomarker/event is uniquely addressed by the test system.

^a^ Both RHuA and hPCLS, offer activated macrophage detection, however, hPCLS, immune cells are already present in the tissue at the time of culture (same donor) and in a spatial context (e.g., intra-alveolar vs interstitial location). RHuA tissue can be supplemented with immune cells (typically from a different donor) after tissue maturation and prior to experimental use. It is unclear how effects of immune cells from a different donor impact the RHuA tissue response/performance during experimentation.

^b^ hPCLS, offer a native tissue architecture, including respiratory parenchyma and pulmonary vasculature. This environment allows the observation of histological changes in a spatial context (e.g., alveolar vs small airway response) where the presence of specific cell types may indicate changes reflective of disease progression and/or hallmark KE (e.g., interstitial collagen deposition). When generated as co-cultures, RHuA can inform on some aspects of tissue architecture, but in a more limited scope than hPCLS.

^c^ hPCLS, can contain airways with beating cilia and whose beat frequency can be quantified. However, due to the infrequent presence of cilia-bearing airways, efforts for this endpoint assay in hPCLS, would likely be low-throughput.

^d^ Exposures conducted using RHuA under ALI, conditions most closely reflect the dosimetry of *in vivo* exposures in represented cell types. Since hPCLS, are generated by slicing lung tissue, the luminal-albuminal compartmentalization is disrupted. This means that there is no longer an epithelial barrier between the test article and underlying non-epithelial cell types (e.g., fibroblasts, endothelial cells, *et cetera*). However, the impact of a disrupted epithelial barrier and whether assay endpoint results are adversely impacted through the loss of this barrier has not been investigated.

### 5.2 Outstanding questions and considerations for a tiered framework

Workshop participants discussed the amount and type of data needed to establish confidence in the utility of a NAM. There was broad agreement on the need to ground any tiered framework in mechanistic considerations. As understanding of the mechanisms of respiratory irritation improves, mechanistic considerations may be grounded in AOPs, developed into an IATA, or ultimately incorporated into a DA, or some other option. Although there was general agreement on the utility of AOPs for setting biological context for NAMs, there were different perspectives regarding the details. Some participants suggested that a more complete AOP is better but left open the possibility that a “complete enough” AOP would still be useful (and left open the definition of “complete enough”). Others focused on the integration of NAM data with other data (i.e., *in silico* or existing *in vivo* data) in a systematic approach leading to a coherent conceptual model in the form of an IATA (Clippinger et al., 2018a; van der Zalm et al., 2022), regardless of the completeness (or even existence) of an AOP. A conceptual model would aid in understanding how particular NAM-based data may or may not be able to inform the understanding of a chemical’s potential hazard. An AOP (or multiple AOPs) provides one way to inform such a conceptual model, but the lack of a well-defined AOP should not prevent the development of this coherent conceptual model. Indeed, a key purpose of AOPs is to provide a structure for organizing the data (Villeneuve et al., 2014a; Villeneuve et al., 2014b). Some participants also noted that AOPs are living documents and are thus never considered fully complete (Villeneuve et al., 2014a).

There was general agreement among participants that any tiered approach should rely on a customizable battery of tests, rather than a single *in vitro* cell/tissue test system that is tuned perfectly for just one test/mechanism/endpoint or one set of physicochemical properties. Any test or set of tests that is too narrow in focus on mechanism or endpoint could miss key markers of irritation. A battery of tests can account for the strengths and weaknesses of different tests and capture a more complete biological picture. From an industry perspective, a requirement for multiple tests could make a testing program more challenging, though this could be mitigated somewhat by development of an IATA and specific DA aimed at KEs. For example, to assess respiratory irritation, one of the biological effects to consider is secretion of inflammatory markers. Models such as ImmuLung ^TM^, ImmuPHAGE ^TM^, ALIsens^®^, and others (Chary et al., 2019; Marescotti et al., 2019) include macrophages (albeit from different donors than the other cell lines in the model), and allow measurement of inflammatory biomarkers (Cook et al., 2019). A participant noted that secretion of inflammatory markers by epithelial cells would also be of interest. Depending on the purpose of the study, one way to build a relevant battery would be to identify the most comprehensive ALI test system that can evaluate as many endpoints as possible, including inflammation markers. After identifying the ALI test system that fits the purpose of the study, an approach could then be built around it.

Participants discussed that a tiered approach could be structured in several different ways. One possibility is as a flow diagram or a decision tree, where one key event from an AOP is tested, and if the test result is positive, the testing proceeds to the next key event. Another option would be a structure similar to the “2 out of 3” KEs approach used for the skin sensitization DA, wherein a certain number of KEs with positive hits results in a “flag” for sensitization (OECD, 2023). These may not be the only ways of structuring a tiered approach. It is also possible that rules for proceeding between different tiers of a tiered approach could be structured in different ways.

Another decision point is whether to focus on inexpensive test systems with limited capabilities but higher throughput or more complex test systems addressing multiple endpoints with greater biological fidelity but lower throughput. These alternatives are not necessarily mutually exclusive but may reflect the need to answer specific questions and have different priorities across stages of the process or for different assessment purposes. Essential to answering this question is identifying which KEs can be assessed relatively inexpensively and quickly, and then using the data generated from these initial tests to inform the next steps. For example, in an initial screening test it may make sense to use tests that are relatively easy to perform and produce reproducible results to obtain a go/no-go decision for ingredients/formulations, while later tiers might use more complex *in vitro* cell/tissue test systems.

There are hundreds of thousands of possible ingredients in cleaning products, and many potential ways of formulating cleaning products. While some contexts (e.g., a regulatory context for a specific formulation) may require testing of only a small number of combinations of possible cleaning product formulations, others (e.g., an early screening effort by a chemical manufacturer for determining ingredients for use in a product) may require testing of a larger number of combinations of ingredients. As such, high (er)-throughput methods would be of benefit in screening, for example by harnessing high throughput transcriptomic methods to identify concentrations that alter relevant biological pathways (Harrill et al., 2019; Harrill et al., 2021a). Use of a transcriptomic approach would add the need for standardization of transcriptomic signatures considered indicative of respiratory irritation. For transcriptomic signatures, a time course is a key variable to consider. The potential for evaluating multiple alternative formulations in a higher throughput format is one potential strength of *in vitro* testing (Price et al., 2020). There is ongoing progress toward an OECD reporting framework for transcriptomics data use in regulatory toxicology (Harrill et al., 2021b) that should also be considered in any framework for identifying respiratory irritation by cleaning products; similar reporting requirements may also be relevant for other high (er)-throughput methods. Some participants noted that increasing throughput can also limit the number of endpoints that can be investigated, so the applicability may vary by purpose.

There are also considerations related to cell culture plate dimensions that pose a challenge to utilization of higher throughput methods. Increasing the number of wells also means decreasing the size of the wells (and thus the mass of the cells/tissues each well can hold, or “biological material”). Thus, one of the disadvantages of using a format with more wells is that the degree of possible multiplexing *can be* reduced (i.e., reducing the variety or number of assay endpoints that can be evaluated with material from a single well). This may be mitigated in some cases by increases in the total biological material per plate (instead of per well) when more wells are added, but this increase is not universal. The relationship between well size and number of wells per plate, and its implications for assay scale, multiplexing, and throughput, should be considered on a case-by-case basis. Further, there are edge effects related to mucociliary phenotype when culturing reconstituted tissues in wells, where mucociliary phenotype is not maintained close to the edge of culture wells. The participants’ opinion was that at this time, culturing in 96-well plates while retaining mucociliary phenotype is possible, but such cultures in 384-well plates primarily consist of phenotypes altered by these edge effects. More research is needed to fully understand the extent of edge effects. As such, different purposes may have a different optimal choice of well size, driven by a balance between throughput and assay scale/multiplexing.

## 6 Applications to human health risk assessment

### 6.1 Importance of NAMs evidence integration with other data in systematic approaches

As noted in the Introduction (Section 1), respiratory irritation was defined for this workshop as disruption of the ELF or epithelial perturbation, but even this limited definition includes challenging biological complexities. Therefore, broader biological understanding of respiratory irritation will be critical in validating and creating context for *in vitro* data, though the specifics of exposure in clinical data may be unknown. These data should be integrated in a systematic manner, leading to a coherent conceptual model in the form of an IATA. Clear documentation of the methods followed to obtain the *in vitro* data is essential to developing confidence in the results and their reproducibility.

The translation between clinical and *in vivo* data to *in vitro* data should be based on mechanistic understanding (informed where possible by KEs and KERs in an AOP) relevant to both physicochemical properties and respiratory irritation. A key challenge will be quantifying the KERs, such as defining how much cytotoxicity results in compensatory cell proliferation. Another key concern of participants in this session was defining criteria for a positive signal in *vitro* assays. Some suggested approaches were to compare results with those obtained with a reference chemical or spike a product with a known respiratory irritant.

There was a consensus in the human health risk assessment (HHRA) breakout group that participants would not be comfortable using *in vitro* ^6^ results alone for respiratory irritation risk assessment, but many would use such data for screening or prioritization or as part of the WOE, if the context of use is clear. For example, using *in vitro* assays in an early tier for screening, and followed up by more traditional tests in a tiered approach might be more acceptable for certain purposes (e.g., regulatory submissions, exposure limit derivation), while such follow up testing might not be needed for internal assessment purposes. Some participants felt that benchmarking the *in vitro* assay results relative to more traditional tests may boost acceptance among users and stakeholders. This could be accomplished by conducting an *in vitro* assay for a chemical with existing animal data as has been done historically. Others stated that the current state of the science is that direct concordance of data from human cell-based *in vitro* assays and animal data should not be expected, and any such analysis should be conducted with caution (Van der Zalm et al., 2022). Biological understanding of the processes leading to respiratory irritation represented by direct cytotoxicity was important in the development of a case study on an IATA for using NAMs to refine an inhalation risk assessment for respiratory irritation (OECD, 2022). Importantly, a FIFRA scientific advisory panel (SAP) reviewing a case study of an *in vitro* approach for refining a pesticide inhalation risk assessment indicated several limitations with the use of cytotoxicity to represent respiratory irritation (U.S. EPA, 2019a). The report also highlighted the need for more data linking effects observed in *in vitro* systems with *in vivo* exposure outcomes.

### 6.2 Dosimetry

Consideration of dosimetry will be critical for applying *in vitro* methods to HHRA, including benchmarking the *in vitro* test conditions to exposure in the *in vivo* use scenario. As described in this section (Section 6.2), choice of the appropriate dose metric is determined based on the chemical’s physicochemical characteristics and the mechanistic step (e.g., MIE or KE) that is the basis for the assay. This dose metric is then used to calculate the human equivalent concentration (HEC), the concentration in air that would result in the same dose to the target as was delivered in the *in vitro* cell/tissue test system of interest, using *in vitro* to *in vivo* extrapolation (IVIVE).

As noted in prior sections (Sections 3, 4), it is important to distinguish the exposure concentration in air or liquid from the internal dose achieved in the assay. The “target site exposure” in the assay can refer to deposited or retained dose at the tissue, cellular, or molecular level. The best dose measures (“metrics”) show a correlation with toxicity, or at least with the response in the KE/endpoint being measured. The definition of dose will be key for appropriate dosimetry. When choosing a dose metric for use in the *in vitro* assay, the scale of the metric should be the same as the observation or the KE used (NASEM, 2017). Internal dose is strongly preferred over external exposure whenever possible. Typically, the internal inhaled dose will be what is delivered to the epithelium, or preferably, the intracellular dose, similar to that used in the *in vivo* assays. This can be normalized to surface area, or, if the MOA or other evidence points to a specific effector cell, dose could be normalized to the number of effector cells (e.g., the number of alveolar macrophages). More generally, for effects in tissues, dose could be based on how many cells are in that tissue; for cellular effects, the dose metric should be intracellular dose.

**FIGURE 4:**
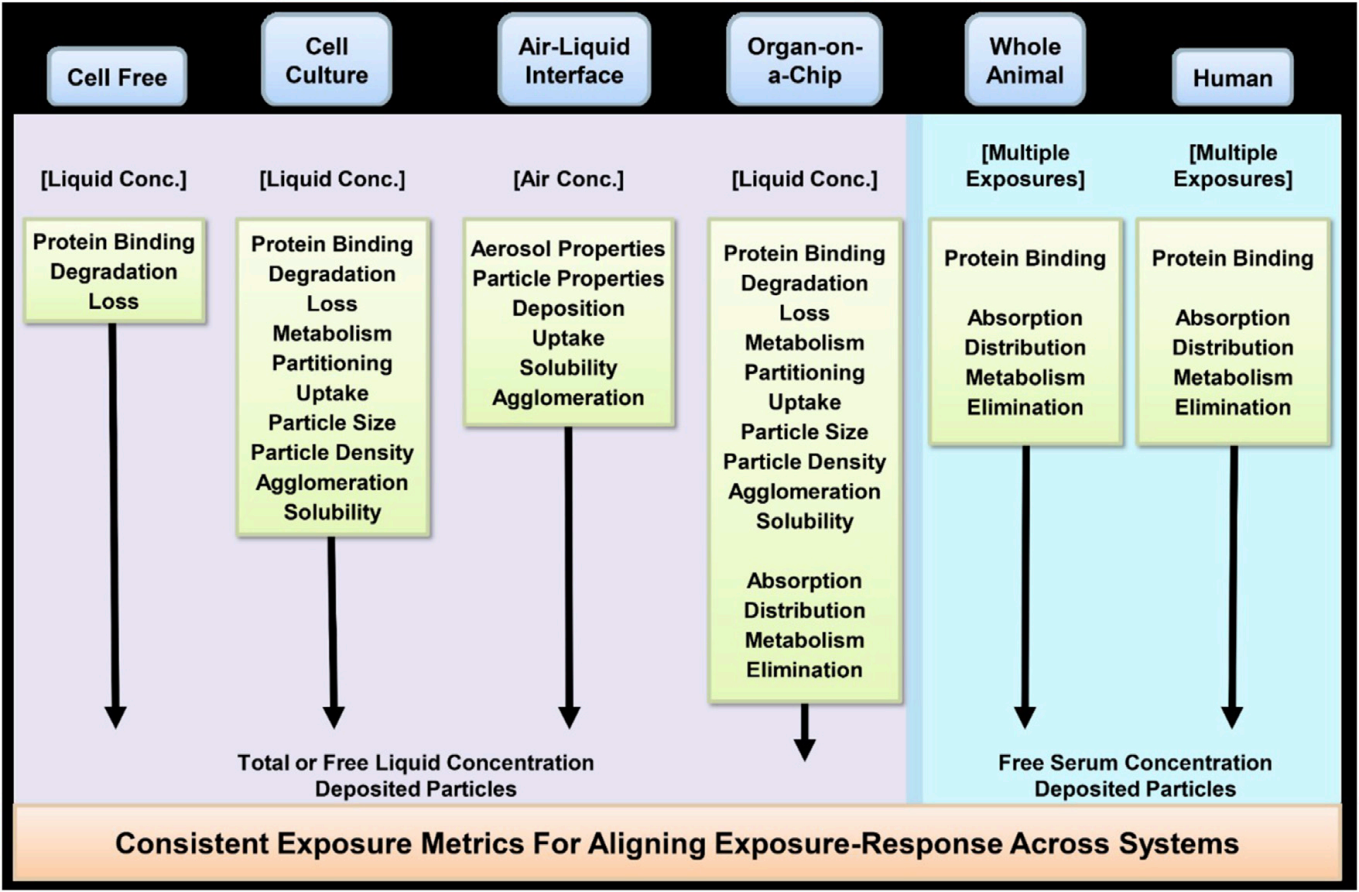
Chemical characteristics, experimental parameters, and mechanistic processes that affect delivered dose. (NASEM, 2017). Note that in 2017, at the time of the NASEM report, lung on a chip used liquid exposure, but advances since then mean that lung on a chip is now more frequently done at ALI, so air concentration would be the exposure metric. In addition, note that “particle properties” includes particle size distribution, and this characteristic is among those determining deposition, and therefore absorption, in the whole animal and human systems.

The goal of the dosimetric adjustment is to integrate *in vivo* and *in vitro* data to systematically account for how physicochemical properties (e.g., particle size, solubility) and product form (e.g., spray, mist, or fog) interact with differences in exposure systems or exposure regimen, respiratory tract anatomy, and physiology (e.g., breathing mode and ventilation activity pattern) to determine the “target site exposure.” Depending on the cell/tissue test system, a variety of different parameters and processes determine the relevant target site exposure. These include protein binding, degradation, metabolism, partitioning, particle size and density, and solubility for cell cultures, and aerosol properties, deposition, uptake, solubility, and agglomeration for ALI systems that utilize cloud rather than liquid exposures (see Figure 4). These various processes mean that the amount of chemical added to a cell or tissue culture does not necessarily directly translate to a dose delivered to the cell/tissue. Thus, while the target site exposure typically is not measured, it is important for coherent evidence integration across experimental platforms in the calculation of the HEC to account for differences between the amount of chemical added to the culture and the actual target site exposure. The same deposition mechanisms occur in air and in liquid media and can be addressed quantitatively. It is also necessary to account for the viscosity of fluid, and whether the fluid is flowing or static. All these elements affect the *in vitro* toxicokinetics (TK) and can factor into the uncertainty factors selected (see Section 6.4).

Characterizing and reporting the determinants of dose will also be important. The choice of dose metric is informed by the level of biological organization of KE (e.g., molecular, cellular). It is possible to select any dose-response relationship along an AOP, based on where there is the most confidence in the causal relationship of KE to adverse outcome (AO). Alternatively, the specific KEs may not be known, but risk assessors will still want to know the internal/intracellular dose. This dose then needs to be scaled appropriately to the HEC based on the dose metric of choice, such as the delivered or intracellular dose per unit area for cytotoxicity related to the internal inhaled dose of the relevant region of the respiratory tract and its surface area. CFD or CFPD models can refine these relationships to localized cell types if relevant.

The choice of specific dosimetry model will be determined by the *in vitro* cell/tissue test system and the physicochemical properties of the test article. The *in vitro* sedimentation, diffusion, and dosimetry model (ISDD; Hinderliter et al., 2010) and *in vitro* sedimentation, diffusion, dissolution, and dosimetry model (ISD3; Thomas et al., 2018) are for submerged test systems; the latter includes dissolution (e.g., for when the ion is the toxic form). CFD or CFPD models can predict localized flux or localized particle deposition from *in vivo* gas or aerosol exposures (Corley et al., 2015; Corley et al., 2021; Lee et al., 2022). Physiologically based pharmacokinetic (PBPK) models are useful for considering systemic dose, and can address metabolism (e.g., Tan et al. (2020), OECD (2023)). Hybrid CFD-PBPK models can address respiratory tract tissue metabolism (Schroeter et al., 2006) and predict localized internal doses such as the area under the curve for parent compound or various metabolites in specific regions. The MPPD model can be used to predict inhaled particle deposition and clearance to estimate retained mass in humans, rats, and mice (ARA (Applied Research Associates), 2024). The U.S. EPA has developed its own version of the MPPD model (U.S. EPA MPPD v2.0 2024) that underwent external expert peer review; it is scheduled for release in 2024. In addition to the capabilities of the version of MPPD released by Applied Research Associates (ARA), the U.S. EPA version will be able to address clearance in the head, has revised clearance rates using more data for rodents (rats and mice), and includes adjustment capabilities for hygroscopicity and solubility. Participants noted that at this time, there are not yet any adequate dosimetry models for ALI exposure systems.

Participants identified several uncertainties around dosimetry and *in vitro* cell/tissue test systems for respiratory irritation. One uncertainty was the impact of a lack of clearance *in vitro*. There was discussion, but no resolution, regarding whether *in vitro* test systems can be considered a worst-case scenario in the absence of clearance. For example, retained dose represents an accumulation over time that does not occur *in vitro*. Participants also questioned whether cumulative dose considerations would apply in the absence of clearance in *vitro* test systems. Another uncertainty was the impact of washing and recovery in the *in vitro* test systems. Conversely, the *in vitro* test system may not have the appropriate enzymes for metabolism, depending on the cell types used (e.g., carboxylesterase is highest in nasal tissue and lower in the lower respiratory tract), though metabolism might be less important for direct contact irritation, which is often related to reactivity of the parent chemical. Another consideration is that, while *in vitro* respiratory irritation models are frequently applicable only to the upper or conducting airway (e.g., use bronchial epithelial cells), *in vitro* respiratory irritation models should also consider the lower airway. Some participants in the broader workshop suggested some of this can be handled with the application of uncertainty factors (UFs); other participants suggested that UFs are an acceptable default in the absence of additional information, but that further characterizing uncertainty or variability might be preferred for some purposes. Using *in vitro* cell/tissue test systems to characterize variability was discussed in Section 4.2. Finally, uncertainties remain regarding other aspects affecting the dose, such as how to account qualitatively and quantitatively for the test article binding with the plate equipment.

### 6.3 Identifying the point of departure for dose-response analysis

Benchmark dose (BMD) modeling is preferred over the No Observed Adverse Effect Level/Lowest Observed Adverse Effect Level (NOAEL/LOAEL) approach to dose-response analysis, because the latter is dependent on study design and doses chosen (U.S. EPA, 2012; EFSA Scientific Committee et al., 2022). In standard BMD modeling, each relevant endpoint is modeled separately, with the effect level based on the benchmark response (BMR), a predetermined change in the level of response. The TRV^7^ or exposure limit is then based on determining the most sensitive relevant BMD (or, typically, the 95% lower confidence limit on the BMD, the BMDL) and dividing this point of departure (POD) by appropriate UFs. Thus, there are three key decision points in deriving TRVs: (1) identifying the appropriate degree of change for the BMR, (2) choosing the appropriate mathematical model(s), and (3) applying appropriate UFs. The first two decision points are discussed in this section (Section 6.3), while UFs are discussed in Section 6.4. As described in the previous section (Section 6.2), dosimetric adjustments or other adjustments to the POD may also be needed to match the experimental exposure conditions and physiology/biology to the exposure scenario of interest (United States Environmental Protection Agency, 1994; NASEM, 2017).

There was a general agreement among participants in the HHRA breakout session that there is a need to evaluate the visual fit to the data from multiple mathematical models and statistically demonstrate goodness of fit, though the specifics of how this should be done were not discussed. One possibility is based on the U.S. EPA BMD guidance: run three different statistical models on each endpoint, demonstrate goodness of fit, and provide the rationale for choice of model, including consideration of the Akaike Information Criterion (AIC) (U.S. EPA, 2012). Alternatively, one could run all relevant models available in the software being used.

The choice of the appropriate BMR (which ultimately determines the POD) is challenging (Haber et al., 2018). There is a lot of uncertainty in defining adverse responses *in vitro,* as well as uncertainty related to technical issues in the assays. It is necessary to understand what causes variability in responses *in vitro* to determine the appropriate BMR. This necessity was discussed in more detail in Section 4. In making this choice, there are a few options and issues to consider. The BMR could be based on a change of one standard deviation from the mean of the reference group or it could be related to the degree of change considered adverse (related to severity). The BMR is likely to be different for different endpoints. Many questions remain for how to systematize an approach to choosing a POD for *in vitro* assays. Some participants noted the use of a BMR of a one standard deviation change in the control mean in the OECD (2022) case study, while others questioned whether this choice was adequately supported. Several recent publications discuss methods of integrating and using transcriptomic data for regulatory purposes (Harrill et al., 2021a; Harrill et al., 2021b; Harrill et al., 2019). Other recent publications that discuss ways of conducting BMD modeling across various assays, often in the context of transcriptomic data (e.g., Speen et al. (2022), Drake et al. (2023)), may also provide insights into how to conduct BMD modeling across multiple assays in a framework like the one above, including evaluation of their relationship to the *in vivo* POD. The need for quantitative AOP (qAOP) has been emphasized to help improve IVIVE and integrate exposure and key events in support of computational modeling and risk assessment (Perkins et al., 2019).

Participants highlighted the importance of standards for reporting data and analysis methods, echoing the importance of GLP and GIVIMP highlighted earlier.

### 6.4 Uncertainty factors

Workshop participants considered the “classical” UFs used with *in vivo* studies to provide an appropriate background on the use of *in vitro* data in HHRA. Although different organizations vary in the details of their approach to UFs, UFs related to human variability (UF_H_) and interspecies extrapolation (UF_A_) are considered by most organizations in standard risk assessments using *in vivo* data. Both the interspecies and intraspecies variability includes uncertainty due to data deficiencies and variability with respect to TK and toxicodynamics (TD). Considerations include the nature of the dosimetry adjustment (e.g., default or optimal model) and the need to account for variability of susceptible populations due to differences in life stage, disease state, and other features. The U.S. EPA also considers UFs related to (1) severity or nature of the endpoint, or LOAEL to NOAEL extrapolation (UF_L_), (2) duration extrapolation (UF_S_), and (3) database uncertainties (UF_D_), typically related to comprehensiveness of the foundational data and questions of whether the appropriate critical effect has been identified (United States Environmental Protection Agency, 1994; U.S. EPA, 2002). Other organizations may use different UFs but consider the same general areas of uncertainty (Haber et al., 2012). Where a specific regulatory organization has jurisdiction, it is important to follow the approach of that organization. Regardless of the approach used, documenting the rationale for the choice of each UF provides needed transparency.

The U.S. EPA and other organizations partition the interspecies and intraspecies UFs into TK and TD components, where chemical-specific data can replace the default values for any of the subfactors (e.g., intraspecies variability in TK) (IPCS, 2005; U.S. EPA, 2014). Such partitioning for intraspecies variability can provide an incentive to address the key questions needed to apply *in vitro* data in HHRA. The discussion below presents perspectives on both use of default approaches and on how available data can replace defaults. Van der Zalm et al. (2022) addressed uncertainties and issues needed to establish scientific confidence in NAMs more broadly, which largely apply to NAMs for respiratory irritation here. They highlight the importance of fitness-for-purpose, reliability, relevance to human biology, independent review, and transparent communication. The text below expands on this approach.

#### 6.4.1 UF_A_ – interspecies extrapolation

Interspecies extrapolation typically addresses TK and TD considerations to account for the differences between animals and humans. The use of human cells or cell lines for *in vitro* assays negates the need for interspecies extrapolation. However, some workshop participants considered a new *in vitro* UF (discussed below in Section 6.4.5) as analogous to the extrapolations involved with applying interspecies UF (e.g., the degree of dosimetry adjustment to characterize the TK determinants of the exposure system, and the reliability and utility of the cell/tissue model as relevant to the *in vivo* target tissue).

#### 6.4.2 UF_H_ – intraspecies variability

Participants agreed that it is useful to continue to divide this UF into default subfactors of 3 each for TK and TD. These subfactors reflect the need to address population variability in ADME and in response (TD) with cell/tissue models from a small number of individuals. Other related uncertainties include consideration of whether the human cells are from immortalized cell lines or primary cells, as well as the adequacy of the number of replicates, and the resulting impact on estimates of central tendency and variability. It was noted that chemical-specific data can replace the default subfactors, and determining the primary causes of variability can help to refine the subfactors.

Understanding population variability in the dose metric of interest for a given exposure level can address intraspecies TK, and donor variability in the response for a given dose (using the appropriate dose metric) addresses intraspecies TD (e.g., McCullough et al. (2016), Bowers et al. (2018), Bowers et al. (2021)). In using donor samples to characterize population variability (Section 4.2) and replace the default factor for intraspecies TD, it would be important to test an adequate sample size, and to ensure that potential sensitive populations are included. It should also be noted that total variability reflects a combination of sampling variability, technical variability, and population variability (Rusyn et al., 2022). It will also be critical to demonstrate that inter-individual variability in exposure outcomes in primary cell-based respiratory tract models is a biological characteristic that is reproducible over time and not just the cumulative impact of technical variation across different exposure experiments. Note that the individual subfactors are not intended to capture the entire range of population variability. Rather, they reflect the range from a measure of central tendency (e.g., the mean) to a high percentile (e.g., 95^th^) on the sensitive end of the distribution (IPCS, 2005; U.S. EPA, 2014). Another perspective, based on a case study on use of an IATA for a respiratory irritant, was that variability in ADME was likely to have minimal impact for a direct-acting irritant, and so the UF for intraspecies TK could be reduced to 1 (OECD, 2022). These two perspectives are not necessarily mutually exclusive, though there may be disagreements about precisely when it is appropriate to reduce UF_H_.

#### 6.4.3 UF_L_ – severity

UF_L_ is usually set to 1 with the use of a BMDL (Haber et al., 2018). Participants discussed uncertainty in how this should be handled in relation to the challenge of selecting appropriate BMR levels for *in vitro* assays. As noted in Section 6.3, it is not clear what an *in vitro* BMR should be, and the appropriate BMR may vary by assay. Some of the early literature on choice of BMR for dichotomous and continuous *in vivo* endpoints may inform such decisions for *in vitro* endpoints (Faustman et al., 1994; Allen et al., 1994a; Allen et al., 1994b; Kavlock et al., 1995).

#### 6.4.4 UF_S_ – duration (classically, subchronic to chronic)

Participants generally agreed that the UF_S_ should be 1 if the duration of assay is comparable to the duration indicated in the problem formation. Depending on the MOA and whether response increases with exposure duration, UF_S_ may need to be larger than 1 for repeated and/or episodic exposures. For example, mild irritation may be driven by concentration, not cumulative exposure, while irritation that results in tissue damage and remodeling increases with exposure duration. The exact magnitude of UF_S_ for various endpoints remains to be decided.

#### 6.4.5 UF_IV_ – *in Vitro* UF

Some participants in the HHRA breakout proposed a new *in vitro* UF to address the uncertainty of using *in vitro* testing as surrogate for *in vivo* testing. In part, this new UF is analogous to the interspecies UF and would need to reflect both TK and TD uncertainties related to IVIVE. Additionally, this UF would address other uncertainties, such as the connections between KE and AO, and how well an *in vitro* cell/tissue test system can represent *in vivo* human response. There was some disagreement among the broader workshop participants about the necessity of the *in vitro* UF.

Those in favor of this new UF suggested it could be modeled after the interspecies UF, with default subfactors of 3 each for TK and TD uncertainties. The TK subfactor would address uncertainties in differences between *in vitro* and *in vivo* dosimetry, as well as the quality of the dosimetry adjustment applied, while the TD factor would address differences in response. Areas of uncertainty and variability specific to *in vitro* systems include issues related to the choice of target system surrogate, target tissue specificity and variability, spatial representation and variability, and metabolic competence and variability. Specific TK considerations include issues related to (1) the *in vitro* delivery mechanisms, (2) well and insert sizes, (3) reactivity of the generation/exposure system with physicochemical properties of the test substance, (4) relevance to target exposure scenario, and (5) cell/tissue system specifications. TD considerations include issues related to consideration of (1) the intended application of the assay relative to the effect or KE it represents, (2) the degree of verification of the assay; (3) target response level (BMR); and (4) existence of performance standards.

Appropriate data could be used to modify default subfactors when available. For example, a submerged cell culture with ISDD addresses dosimetry, and thus the *in vitro* TK is addressed. Similarly, if CFD is used to perform IVIVE for an ALI exposure with appropriate scale up to an HEC, it may not be necessary to apply additional dosimetry adjustments. However, it would be necessary in these cases to characterize the determinants of dose or to recognize that as an uncertainty. In addition, the TD component would need to be addressed by how well the *in vitro* test system captures issues of susceptible populations.

Those opposed to an *in vitro* UF noted that the OECD (2022) case study did not include an *in vitro* UF. They argued that other aspects of *in vitro* testing, such as the absence of clearance, compensate for the IVIVE, and thus they believe that an *in vitro* uncertainty factor would not be required. Others felt that the absence of biologically-relevant ADME including clearance processes (M and E) *increases* uncertainty compared to an *in vivo* experiment. This premise raised the issue of whether *in vitro* cell/tissue test systems are always conservative from the dose perspective. Exposure is dynamic *in vivo*. This dynamic aspect is not reflected *in vitro,* but other aspects of dosimetry are often not well-characterized. For example, *in vitro*, the chemical is often sticking to the plate, meaning that the delivered dose is less than expected based on the applied dose.

The question was also raised about how well a single assay, or even a battery of assays, can address the respiratory tract as a system. In addition to uncertainties about the respiratory tract, uncertainties about systemic effects were also noted. Such uncertainties could be part of this UF or UF_D_, but such issues were beyond the scope of the workshop.

#### 6.4.6 UF_D_ - database

The database uncertainty factor addresses whether the correct endpoint is being considered (i.e., whether the NAM provided comprehensive coverage of potential toxicity). In other words, UF_D_ addresses whether all of the assays conducted provide adequate confidence that all relevant potential effects have been considered and the appropriate endpoint is being used for the POD. A single assay will be unable to do this, although it may be possible with a specified battery of assays. Further, it is not clear how the possibility of systemic effects should be considered.

It is uncertain at this point whether the proposed *in vitro* UF would address coverage, or whether a separate UF_D_ is needed. There is a possibility of overlap between the two if a battery of assays is deployed, so it is not clear whether the two factors should be conceptualized separately. Others felt that the *in vitro* and database factors represent separate areas of extrapolation.

## 7 Conclusion

Although the purpose of this workshop was not necessarily to reach consensus among workshop participants, several key themes emerged from multiple breakout groups. These key themes often led to recommendations for next steps. Common themes included.

• Some of the challenges related to characterizing respiratory irritation are not unique to *in vitro* cell/tissue test systems and are shared with *in vivo* models. These challenges include ensuring intra- and inter-laboratory reproducibility and ensuring that both assays and controls perform as expected. Variability in exposure (both real-world and in experimental systems), *in vitro* test systems, and human populations need to be characterized and understood.
• The nature of the assessment informs the choice of experimental exposure system, the *in vitro* cell/tissue test system and method, and the use of the data to inform human health assessments. Assessments can be categorized as (1) qualitative screening-level hazard identification, (2) industry semi-quantitative or qualitative risk assessment, and (3) deriving a TRV or a quantitative risk assessment conducted for the purposes of regulatory acceptance. The burden of proof increases sequentially across these assessment types.
• The physicochemical properties of the cleaning product ingredient or formulation can guide the choice of both the exposure system and *in vitro* cell/tissue test system. A literature survey/review to identify how the physicochemical properties influence the selection of exposure method and cell/tissue test system is recommended as a key step towards characterization and standardization of exposure and cell/tissue test system methods.
• Building confidence in the use of NAMs requires use of all available data–*in vivo* data from clinical studies and animal data, *in vitro* data, and *in silico* analyses/data–to create context and a better basis for understanding and inference.
• Systematic integration of NAM data with other data (existing *in vivo* data from humans and animals, mechanistic data, *in silico* data, etc.) into a coherent conceptual model or an IATA is critical. The NAMs being evaluated will usually be linked to specific KEs, although a fully described AOP is not necessary.
• Consideration of dosimetry and identification of an appropriate dose metric(s) are essential. It is important to coordinate the choice and measurement of dose metric across the experimental exposure system, *in vitro* cell/tissue system, and quantification approach.
• Characterization of assay methods and standardization of reporting is needed and will facilitate the use of data from assays for a range of applications. Until reporting standards are developed, it is important to document all elements of experimental systems to aid in reproducibility and in determining which design elements have important impacts on the results. It is important to note that exposure concentration for an exposure at ALI is not an appropriate dose metric given that deposition efficiencies vary across ALI exposure systems and are influenced by the physicochemical properties of the test article. Similarly, nominal solution concentration in liquid dosing does not reflect the impact of various factors (e.g., molecular diffusivity, volatility) that influence the exposure of the cells in the *in vitro* system to the test article.
• A tiered approach (such as one like the illustrative example outlined in Section 5.1) is recommended to help make the complex problem of *in vitro* testing for respiratory irritation by cleaning products tractable. Consideration of physicochemical properties, together with *in silico* analyses, is important in early tiers. Further work is needed to develop the tiering, including the specifics of the decision logic.
• Many of the elements of developing risk values from *in vivo* data can be applied to NAMs. For example, BMD modeling is preferred over the NOAEL/LOAEL approach for dose-response analysis, although more research is needed regarding the choice of the BMR.
• The UFs used for *in vivo* assessments can also be adapted for use with NAMs. There was general agreement that the use of human cells removes the need for an interspecies factor, although other aspects of IVIVE need to be considered. Dividing the human variability UF into TK and TD subfactors can provide an incentive for obtaining data to address key questions and replace subfactor defaults with chemical-specific data. Additional research is needed regarding a database UF for adequacy of assay or test battery coverage, and whether a separate UF is needed to address uncertainties related to using *in vitro* testing as a surrogate for *in vivo* testing.
• There is a need for cross-sector collaboration on characterization/standardization and framework development. Engagement from industry, non-governmental organizations (NGOs) and non-profits, consultants, and government agencies will be needed, including those with laboratory expertise and those experienced in the application of such data. This collaboration should consider the different requirements for different types of assessments (e.g., screening versus regulatory-related).
• There will need to be sponsorship and funding for a characterization/standardization effort, from multiple organizations.
• It is important to recognize that buy-in and characterization/standardization requires an ongoing, collaborative effort; a high-quality paper or presentation at a meeting does not automatically lead to standardization without further support.

## Footnotes

1 ACI is a non-profit trade association made up of more than 150 member companies, representing manufacturers of household, industrial, and institutional cleaning products, their ingredients and finished packaging, as well as oleochemical producers, and chemical distributors to the cleaning products industry. https://www.cleaninginstitute.org/

2 The State of California (Breast Cancer Prevention Partners, 2017) defines a general cleaning product as “a soap, detergent, or other chemically formulated consumer product labeled to indicate that the purpose of the product is to clean, disinfect, or otherwise care for fabric, dishes, or other wares; surfaces including, but not limited to, floors, furniture, countertops, showers, and baths; or other hard surfaces, such as stovetops, microwaves, and other appliances.” The U.S. EPA Safer Choice Program for cleaning products addresses products that clean in the following categories: all-purpose, hard surface, glass, degreasers, kitchen and bath, hand dish, drain cleaning and maintenance, floor care, carpet care, car care, laundry, dish detergents, marine cleaning, graffiti removal, and odor removal (U.S. EPA, 2015).

3 Antimicrobials were not the intended focus for this workshop.

4
Integrated Approaches to Testing and Assessment (nih.gov)

5 The respiratory irritation case study (OECD, 2022) discussed later in this report references an AOP, but that AOP is not specific to the respiratory tract.

6 It is recognized that other portions of this report note the importance of considering *in vitro* data together with *in silico* data and AOPs, but the consensus question was framed specifically in terms of use of *in vitro* data. The framing of the question was intended to distinguish between the use of *in vitro* data in the absence of broader biological understanding and the growing opportunities for moving to *in vitro* testing when the underlying biology is well-understood based on prior testing.

7 Note that the BMD is a point of departure that is divided by UFs to result in a TRV, or health “benchmark.”

## Glossary

ACI: American Cleaning Institute
ADME: absorption, distribution, metabolism and elimination
ALI: air-liquid interface
AOP: adverse outcome pathway
BMD: benchmark dose
BMDL: 95% lower confidence limit on the BMD
BMR: benchmark response
CBF: ciliary beat frequency
CFD: computational fluid dynamics
CFPD: computational fluid particle dynamics
2D: two dimensional
3D: three dimensional
DA: defined approach
ECHA: European Chemicals Agency
ECM: extracellular matrix
ELF: epithelial lining fluid
EU: European Union
DA: defined approach
FIFRA: Federal Insecticide, Fungicide and Rodenticide Act
GIVIMP: Guidance Document on Good *In Vitro* Method Practices
GLP: Good Laboratory Practices
GSD: geometric standard deviation
HEC: human equivalent concentration
HHRA: human health risk assessment
hPCLS: human precision cut lung slices
IATA: integrated approach to testing and assessment
ICRP: International Commission on Radiological Protection
ISDD: *in vitro* sedimentation, diffusion, and dosimetry model
IVIVE: *in vitro* to *in vivo* extrapolation
KE: key event
KER: key event relationship
LOAEL: lowest observed adverse effect level
MMAD: mass median aerodynamic diameter
MPPD: multiple-path particle dosimetry
MIE: molecular initiating event
MOA: mode of action
MTT: 3-[4,5-dimethylthiazol-2-yl]-2,5 diphenyl tetrazolium bromide
NAM(s): new approach methodologies
n.d.: no date
NOAEL: no-observed-adverse effect level
NRC: National Research Council
OECD: Organisation for Economic Co-operation and Development
OSHA: Occupational Safety and Health Act
PBPK: physiologically based pharmacokinetic
PET: polyester terephthalate
POD: point of departure
PPE: personal protective equipment
QCM: quartz crystal microbalance
REACH: Registration, Evaluation, Authorisation, and Restriction of Chemicals
RfC: Reference Concentration
RH: relative humidity
RHuA: reconstituted human airways
SOP: standard operating procedure
TD: toxicodynamic(s)
TK: toxicokinetic(s)
TRV: toxicity reference value
TSCA: Toxic Substances Control Act
UF: uncertainty factor
WOE: weight of evidence
