# Supplementary material for: New approach methodologies (NAMs) for the in vitro assessment of cleaning products for respiratory irritation: workshop report
Source: Front Toxicol. 2024 Oct 8;6:1431790. doi: 10.3389/ftox.2024.1431790 (PMC11493779; doi:10.3389/ftox.2024.1431790)
Supplement: Supplementary file 1 [file DataSheet1.PDF]

Supplementary Information

New Approach Methods (NAMs) for the *In Vitro* Assessment of Cleaning Products for Respiratory Irritation
Workshop Agenda - Thursday, March 2, 2023

Product safety is a key component of product stewardship. In the cleaning product industry, manufacturers need tests to assess the hazards of their products to promote safe and responsible use. In this workshop, we will review *in vitro* cellular and tissue-based testing methods for respiratory irritation throughout the life cycle of cleaning products, from raw materials, formulation of intermediate and end use products, and use in their intended markets. The content of the workshop will be used to generate a published Workshop Proceedings Report to assist the cleaning products industry in best practices and principles when selecting testing methods for their products.

8:30 AM - 9:45 AM (EST)
Welcome and General Session

Goals and background for the workshop content will be presented to prepare the audience for breakout discussions.
Topics will include clinical presentation, adverse outcome pathways, animal test limitations, and available new approach methodologies (NAMs) for respiratory irritation.

Speakers include:
Kathryn Page, PhD, *The Clorox Company*
Holger Behrsing, PhD, *Institute for In Vitro Sciences*, Gaithersburg, MD
Shaun McCullough, PhD, *U.S. Environmental Protection Agency*, Chapel Hill, NC
Monita Sharma, PhD, *PETA Science Consortium International*

10:00 AM - 12:00 PM (EST)
Concurrent Sessions

Three Sessions to be held concurrently in separate meeting rooms. Each breakout group will be tasked with addressing a key topic area and have a list of pre-prepared charge questions to discuss.

Concurrent Session A: Exposure Methods

Discussion topic examples:

- Air (aerosol and gas) vs liquid exposures
- Repeat dose vs single exposure.

Moderator:
Kathryn Page, PhD, *The Clorox Company*

Panelists Include:
Vivek Patel, PhD, *Institute for In Vitro Sciences*, Gaithersburg, MD
Clive Roper, PhD, *Roper Toxicology Consulting Limited*, Edinburgh, UK
Shaun McCullough, PhD, *U.S. Environmental Protection Agency*, Chapel Hill, NC

Concurrent Session B: Models

Discussion topic example:

- Single cell vs 3D-tissue vs *ex vivo* (e.g. precision cut lung slices) models

Moderator:
Keith Genco, *Arkema Inc.*, King of Prussia, PA

Panelists Include:
Holger Behrsing, PhD, *Institute for In Vitro Sciences*, Gaithersburg, MD
Sabina Burla, PhD, *Invitrolize;* formerly of *Luxembourg Institute of Science and Technology*, Belvaux, Luxembourg
Phillip Clapp, PhD, *Wake Forest Institute for Regenerative Medicine*, Winston-Salem, NC

Concurrent Session C: Application for Human Health Protection

Discussion topic examples:

- Use of uncertainty factors
- Ingredient concentrations of human relevance
- *In vitro to in vivo* extrapolation (IVIVE)
- Application to human risk assessment for respiratory irritation

Moderators:
Nathan Pechacek, MS, *Ecolab*, Eagan, MN
Casey Fisher, PhD, *Ecolab*, Eagan, MN

Panelists Include:
Scott Dotson, PhD, CIH, *Insight Exposure & Risk Sciences Group*, Cincinnati, OH
Annie M. Jarabek, PhD, *U.S. Environmental Protection Agency*, Research Triangle Park, NC
Monita Sharma, PhD, *PETA Science Consortium International*, Rochester, MI

1:30 PM - 2:30 PM (EST)
Concurrent Sessions - Continued

3:00 PM - 4:45 PM (EST)
General Session: Concurrent Session Reports & Discussion

4:45 PM - 5:00 PM (EST)
General Session: Workshop Summary & Conclusions

Workshop Charge Questions
New Approach Methods (NAMS) for the *In Vitro*
Assessment of Cleaning Products for Respiratory Irritation

1 Session A: Exposure Methods

1.1 Existing Guidance

a. What criteria/approach(es)/framework(s) should be used to evaluate the appropriateness, relevance, and adequacy of an exposure method for a given application? Suggested frameworks/considerations include, but are not limited to the following. Short form references are given here, and full references in a reference list at the end:

   i. OECD 2018 good *in vitro* methods and practices

   ii. Petersen et al. 2022. Framework for high quality measurements in NAMs

   iii. Whalan et al. 2019. Evaluation of inhalation studies for exposure quality.

   iv. Clippinger et al. 2018. Pathway-based approaches for non-animal assessment of acute inhalation hazard determination.

   v. ARRIVE guidelines. Animal research. Reporting of *in vivo* experiments. (These are *in vivo,* but form the basis for the RIVER standards under development.)

   vi. Do any relevant regulatory agencies have related criteria/approach(es)/framework(s) that should especially be considered?

1.2 Characterizing the exposure system:

a. Biological Relevance

   i. What are relevant exposure regimens (e.g., single 6-hr, daily for 5 days) and durations (e.g., two weeks) to capture relevant responses? How do these vary depending on the assay?

   ii. What should be considered in the selection of cell type (e.g., nasal versus pulmonary)?

   iii. Are epithelial-only, or other monoculture, *in vitro* systems a suitable representation of their respective regions of the respiratory tract *in vivo*? (see Faber et al., 2020 in reference list)

   iv. Are the determinants of internal dose described for the exposure system?

v. What are the pros and cons of relying on AOP-directed assay selection versus using an *in vivo* physiology relevant assay battery?

b. Technical Considerations

i. Are there “watch-outs” for exposure systems based on physicochemical properties?

1. What are the implications of using air (aerosol or gas) exposures compared with liquid (submerged or direct application) exposures? (e.g., see Mallek et al., preprint in reference list)

2. Do the physicochemical properties of the material being tested or the type of cell system (e.g., primary cell, 3D explant) affect the choice/appropriateness of different exposure systems? If so, how?

ii. What parameters are needed to adequately characterize exposure (e.g., temperature, relative humidity, air flow, volume of liquid, well size and material, nominal versus delivered concentration)? (E.g., see Whalan et al. 2019.)

iii. What tests are necessary to demonstrate lack of reactivity with the exposure system?

iv. For how long do the respective exposure systems maintain a consistent exposure concentration?

v. What is the impact of liquid dosing or washing on the integrity of the cell culture?

vi. What information needs to be considered in the interpretation of commonly used assays and endpoints?

1.3 Exposure Levels and Dosimetry.

a. How should the choice of dose metric be informed by the physicochemical properties and target use application?

b. How should the experimental exposure levels be chosen?

4. What level of validation or evaluation approach would be suitable for regulatory acceptance? How is this different for screening applications vs. development of health benchmarks?

5. What are key references that should be cited in the manuscript?

2 Session B: *In Vitro* Models

2.1 Existing Guidance

a. What criteria/approach(es)/framework(s) (e.g.,) should be used to evaluate the appropriateness, relevance, and adequacy of an *in vitro* cell test system for a given application? Suggested frameworks/considerations include, but are not limited to the following. Short form references are given here, and full references in a reference list at the end:

   i. OECD 2018 good *in vitro* methods and practices

   ii. Petersen et al. 2022. Framework for high quality measurements in NAMs

   iii. Whalan et al. 2019. Evaluation of inhalation studies for exposure quality.

   iv. Clippinger et al. 2018. Pathway-based approaches for non-animal assessment of acute inhalation hazard determination.

   v. ARRIVE guidelines. Animal research. Reporting of *in vivo* experiments. (These are *in vivo*, but form the basis for the RIVER standards that are under development.)

   vi. Do any relevant regulatory agencies have related criteria/approach(es)/framework(s) that should especially be considered?

2.2 Test System, Test Conditions, and Relevance

a. Of the variety of available test systems (e.g., cells, 3D explant systems, precision-cut lung slices [PCLS], single cell type vs. coculture), which are preferred features for which assays and why?

   i. Are airway epithelial cell lines, cells with inherently abnormal characteristics (highly proliferative cells that cannot differentiate to a normal/stable mucociliary phenotype) suitable for assessing inhalation toxicity? Are models with human primary cells better? Does this vary by assay and why?

   ii. As *in vitro* model systems continue to emerge and evolve, they are often promoted as “enhanced”, “improved”, or “more physiologically relevant”. How do we confirm the accuracy of these claims? How complex or “organotypic” does a model need to be for inhalation toxicity studies?

b. For how long do the respective test systems maintain a consistent exposure concentration, viability, and ability to respond to challenge?

i. How can we be confident in an acute assay to characterize the potential for effects after repeated or chronic exposures? (i.e. are specific changes required in order to adapt these assays for repeat-dose exposures?)

c. Adverse outcome pathways (AOP), including molecular initiating events (MIE) and key events (KE), have demonstrated utility in framing directed approaches (DA) for skin sensitization and were recently proposed to help frame similar advances for inhalation exposures (see Figure 2 in Clippinger et al., 2018).

    i. For which KE(s) in the AOP is each cell system (e.g., primary cell, 3D explant, PCLS) accepted or established as relevant?

    ii. Are there other KE(s) that any of these cell systems *could* address, for which relevance has not yet been established?

d. What performance measures should be used to evaluate the assay results (e.g., use of reference chemicals or functional measures)?

e. Remodeling and other key events often require recruitment of immunocompetent cells (e.g., ILC2 or alveolar macrophages). How can this be addressed in the cell system?

f. Are current models representative of susceptible populations (e.g., children or asthmatics) to provide confidence that data resulting from their use would be protective?

2.3 Integrating Across and Beyond Assays

a. What battery (or batteries) of assays would provide confidence regarding coverage of potential KEs and are there any "watch-outs" or "guardrails" for use (e.g., specific to physicochemical properties)?

b. Integrated approaches to testing and assessment (IATA) help to frame evidence integration for risk evaluation. What other data are available to create context for considering these assays? How "complete" does the AOP need to be to establish confidence in the utility of the NAM?

2.4 Dosimetry and Dose-Response Considerations

a. How is intracellular dose (versus exposure concentration) determined in the cell system?

b. Should the choice of dose metric be informed by the physicochemical properties and the target use application?

c. How are response levels established (e.g., anchoring to *in vivo* effects)?

2.5 Nearly all commercially available pulmonary cell lines are from male donors. Should we be addressing sex-specific (and other variable) responses to airborne toxicants? If so, how?

2.6 What are key references that should be cited in the manuscript?

2.7 Supplemental Questions (to be addressed on a time available basis)

a. Which are the best methods and approaches for the assessment of difficult to test chemicals/mixtures?

   i. Which models should be considered to evaluate test chemicals that degrade in the test system?

   ii. Which models for chemicals with low solubility in solvents?

   iii. Which models for volatile chemicals?

   iv. Which models for chemicals that hydrolyze?

   v. Which models for chemicals that undergo oxidation?

   vi. Which models for chemicals that undergo photo-degradation?

   vii. Which models are of choice to assess test chemicals inducing toxicity at low concentrations?

3 Session C: Application for Human Health Protection

3.1 Point of Departure

a. How should NAMs for respiratory irritation be integrated into weight of evidence, systematic risk analysis approaches?

   i. Where can a NAM-based point of departure (POD) be used in place of data from an *in vivo* animal model (i.e., the complete human respiratory tract)?

   ii. For respiratory irritation, how does the relevance of a NAMs-based POD in a human cell line-derived model compare with that from an *in vivo* rodent model?

   iii. Are NAMs-based approaches sufficiently developed to be the sole basis of a human risk assessment for respiratory irritation? If so, are there any “watchouts” or “guardrails” to be considered?

b. Can established guidance for dose-response analysis be applied to *in vitro* systems (e.g., evaluation of various response models, demonstration of goodness of fit (GOF), choice of representative response, etc.)? (See US EPA, 2012, benchmark dose technical guidance)

    i. What adaptations, if any, are needed?

    ii. What precautions should be applied in applying these approaches?

3.2 Dosimetry Considerations

a. How can dosimetric adjustments be incorporated into human health risk assessments using NAMs-based data related to respiratory irritation?

    i. What degree of confidence can be placed in using exposure versus an internal dose measure when conducting *in vitro* to *in vivo* extrapolation (IVIVE)?

    ii. For evidence integration and leveraging all data, multiple types of extrapolations need to be considered: including *in vitro* to *in vivo*, animal to human, and considering human variability. How should each of these extrapolations be considered?

b. What types of dosimetry models can be used to perform IVIVE in the context of respiratory irritation? Are there physiochemical properties, use conditions, or other scenarios, which would impact the choice among the following:

    i. ISDD – *In vitro* Sedimentation, Diffusion and Dosimetry model.

    ii. ISD3 - *In vitro* Sedimentation, Diffusion, Dissolution and Dosimetry.

        1. See https://www.pnnl.gov/projects/vitro-dosimetry for access to both ISDD and ISD3

    iii. CFD - . Computational fluid dynamics. Many relevant publications.

    iv. PBPK – Physiologically based pharmacokinetic modeling. Many guidance documents available from various organizations. E.g., see OECD 2021

    v. MPPD – Multipathway Particle Dosimetry model. Available at https://www.ara.com/mppd/ New US EPA MPPD 2.0 model and documentation out Spring 2023.

3.3 Uncertainty and Uncertainty Factors

a. How can the uncertainty in a NAM-based POD be determined?

i. How should uncertainty in the degree of fidelity (surrogacy) and relevancy to the biological system of the given exposure system and cell model be characterized?

b. What uncertainty factors should be used? Do the conventional areas of extrapolation (TK, TD, duration, severity, coverage) apply?

i. Should a framework be developed to determine appropriate uncertainty factors when NAMs are used as the basis for the POD?

ii. Are additional uncertainty or modifying factors needed for the use of NAMs as the basis for the POD? Specifically, are there other uncertainty factors specific to IVIVE (or rationale for removal of typical ones used, or to justify not requiring additional factors)?

iii. How should uncertainty due to limited replicates or limited number of donors be characterized?

1. How should assessments account for the situation that nearly all commercially available pulmonary cell lines are from male donors (do these apply to the potential for other genetic variability in the population, e.g. those that may exist due to ethnicity)?

iv. Are there published examples or case studies that can be used to strengthen or address these uncertainty factors? See also:

1. Bowers et al., 2021 in reference list

2. Bowers et al., 2018 in reference list

3. OECD 2022 Case study

3.4 Are there considerations that are applicable when we are assessing mixtures vs. single chemicals?

3.5 What are key references that should be cited in the manuscript?

4 References

4.1 References cited in the charge questions

1. ARRIVE guidelines 2.0: updated guidelines for reporting animal research. Originally published in PLOS Biology, July 2020. https://doi-org.uc.idm.oclc.org/10.1371%2Fjournal.pbio.3000410

2. Bowers EC, Martin EM, Jarabek AM, Morgan DS, Smith HJ, Dailey LA, Aungst ER, Diaz-Sanchez D, McCullough SD. Ozone Responsive Gene Expression as a Model for Describing Repeat Exposure Response Trajectories and Interindividual Toxicodynamic Variability In Vitro. Toxicol Sci. 2021 Dec 28;185(1):38-49. doi: 10.1093/toxsci/kfab128. PMID: 34718810; PMCID: PMC8714356. https://doi.org/10.1093/toxsci/kfab128

3. Bowers EC, McCullough SD, Morgan DS, Dailey LA, Diaz-Sanchez D. ERK1/2 and p38 regulate inter-individual variability in ozone-mediated IL-8 gene expression in primary human bronchial epithelial cells. Sci Rep. 2018 Jun 20;8(1):9398. doi: 10.1038/s41598-018-27662-0. PMID: 29925859; PMCID: PMC6010411. https://doi.org/10.1038/s41598-018-27662-0

4. Clippinger AJ, Allen D, Behrsing H, BéruBé KA, Bolger MB, Casey W, DeLorme M, Gaça M, Gehen SC, Glover K, Hayden P, Hinderliter P, Hotchkiss JA, Iskandar A, Keyser B, Luettich K, Ma-Hock L, Maione AG, Makena P, Melbourne J, Milchak L, Ng SP, Paini A, Page K, Patlewicz G, Prieto P, Raabe H, Reinke EN, Roper C, Rose J, Sharma M, Spoo W, Thorne PS, Wilson DM, Jarabek AM. Pathway-based predictive approaches for non-animal assessment of acute inhalation toxicity. Toxicol In Vitro. 2018 Oct;52:131-145. doi: 10.1016/j.tiv.2018.06.009. Epub 2018 Jun 20. PMID: 29908304; PMCID: PMC6760245. https://doi.org/10.1016/j.tiv.2018.06.009

5. Faber SC, McNabb NA, Ariel P, Aungst ER, McCullough SD. Exposure Effects Beyond the Epithelial Barrier: Transepithelial Induction of Oxidative Stress by Diesel Exhaust Particulates in Lung Fibroblasts in an Organotypic Human Airway Model. Toxicol Sci. 2020 Sep 1;177(1):140-155. doi: 10.1093/toxsci/kfaa085. PMID: 32525552; PMCID: PMC8312750. https://doi.org/10.1093/toxsci/kfaa085

6. Henry et al. #2583 | Surfactants Category: An Integrated Approach to Testing and Assessment (IATA) Including New Approach Methods (NAMs) for Assessing Inhalation Risks under the Toxic Substances Control Act (TSCA). US EPA, Washington, DC. https://www.thepsci.eu/wp-content/uploads/2021/03/SOT21_General-Surfactants-Category-Poster-02-March-2021.pdf

7. Hinderliter, P.M., Minard, K.R., Orr, G. *et al.* ISDD: A computational model of particle sedimentation, diffusion and target cell dosimetry for *in vitro* toxicity studies. *Part Fibre Toxicol* **7**, 36 (2010). https://doi.org/10.1186/1743-8977-7-36.

8. Nicholas M. Mallek, Elizabeth M. Martin, Lisa A. Dailey et al. Liquid Application Dosing Alters the Physiology of Air-Liquid Interface Primary Bronchial Epithelial Cultures and In Vitro Testing Relevant Endpoints, 24 February 2023, PREPRINT (Version 1) available at Research Square [https://doi.org/10.21203/rs.3.rs-2570280/v1]

9. OECD. 2018. Guidance Document on Good In Vitro Method Practices (GIVIMP), OECD Series on Testing and Assessment, No. 286, OECD Publishing, Paris. https://doi.org/10.1787/9789264304796-en.

10. OECD. 2021. Guidance document on the characterization, validation, and reporting of Physiologically Based Kinetic (PBK) models for regulatory purposes, OECD Series on Testing and Assessment, No. 331, Environment, Health and Safety, Environment Directorate, OECD. https://www.oecd.org/chemicalsafety/risk-assessment/guidance-document-on-the-characterisation-validation-and-reporting-of-physiologically-based-kinetic-models-for-regulatory-purposes.pdf

11. OECD. 2022. Case Study on the use of an Integrated Approach for Testing and Assessment (IATA) for New Approach Methodology (NAM) for Refining Inhalation Risk Assessment from Point of Contact Toxicity of the Pesticide, Chlorothalonil, OECD Series on Testing and Assessment, No. 367, ENV/CBC/MONO(2022)31, OECD Publishing, Paris. https://www.oecd.org/officialdocuments/publicdisplaydocumentpdf/?cote=ENV/CBC/MONO(2021)35&docLanguage=En [UFs addressed on p. 47]

12. Petersen EJ, Elliott JT, Gordon J, Kleinstreuer NC, Reinke E, Roesslein M, Toman B. Technical framework for enabling high quality measurements in new approach methodologies (NAMs). ALTEX. 2023;40(1):174-186. doi: 10.14573/altex.2205081. Epub 2022 Jul 15. PMID: 35867862. https://doi.org/10.14573/altex.2205081

13. Ramanarayanan T, Szarka A, Flack S, Hinderliter P, Corley R, Charlton A, Pyles S, Wolf D. Application of a new approach method (NAM) for inhalation risk assessment. Regul Toxicol Pharmacol. 2022 Aug;133:105216. doi: 10.1016/j.yrtph.2022.105216. Epub 2022 Jul 8. PMID: 35817205. https://doi-org.uc.idm.oclc.org/10.1016/j.yrtph.2022.105216

14. Tan YM, Chan M, Chukwudebe A, Domoradzki J, Fisher J, Hack CE, Hinderliter P, Hirasawa K, Leonard J, Lumen A, Paini A, Qian H, Ruiz P, Wambaugh J, Zhang F, Embry M. PBPK model reporting template for chemical risk assessment applications. Regul Toxicol Pharmacol. 2020 Aug;115:104691. doi: 10.1016/j.yrtph.2020.104691. Epub 2020 Jun 2. PMID: 32502513; PMCID: PMC8188465. https://doi-org.uc.idm.oclc.org/10.1016/j.yrtph.2020.104691

15. Thomas, D.G., Smith, J.N., Thrall, B.D., Baer, D.R., Jolley, H., Munusamy, P., Kodali, V., Demokritou, P., Cohen, J., Teeguarden, J.G., 2018. ISD3: a particokinetic model for predicting the combined effects of particle sedimentation, diffusion and dissolution on cellular dosimetry for in vitro systems. Particle and Fibre Toxicology 15. https://doi.org/10.1186/s12989-018-0243-7.

16. U.S. EPA MPPD (2023) v.2.0. Multiple-Path Particle Dosimetry (MPPD) Technical Support Documentation and User's Guide. In press.

17. U.S. EPA. 2012. Benchmark Dose Technical Guidance (pdf). Risk Assessment Forum, U.S. Environmental Protection Agency, Washington, DC 20460. EPA/100/R-12/001, June 2012. https://doi.org/10.1016%2Fj.yrtph.2019.104504

18. U.S. EPA. 2018. Issue Paper: Evaluation of a Proposed Approach to Refine Inhalation Risk Assessment for Point of Contact Toxicity: A Case Study Using a New Approach Methodology (NAM). https://www.regulations.gov/docket/EPA-HQ-OPP-2018-0517

19. U.S. EPA. 2018. SAP Evaluation of a proposed approach to refine inhalation risk assessment for point of contact toxicity. https://www.regulations.gov/docket/EPA-HQ-OPP-2018-0517

20. U.S. EPA. 2019. FIFRA Scientific Advisory Panel Meeting Minutes and Final Report No. 2019-01 Peer Review on Evaluation of a Proposed Approach to Refine the Inhalation Risk Assessment for Point of Contact Toxicity: A Case Study Using a New Approach Methodology (NAM). https://www.regulations.gov/docket/EPA-HQ-OPP-2018-0517

21. Whalan JE, Stanek J, Woodall G, Reinhart P, Galizia A, Glenn B, Kraft A, Makris SL, Jarabek AM. The evaluation of inhalation studies for exposure quality: A case study with formaldehyde. Toxicol Lett. 2019 Sep 15;312:167-172. doi: 10.1016/j.toxlet.2019.05.011. Epub 2019 May 14. PMID: 31100492. https://doi-org.uc.idm.oclc.org/10.1016/j.toxlet.2019.05.011

4.2 Key additional references cited in the general presentation talks

1. Carusi A, Davies MR, De Grandis G, Escher BI, Hodges G, Leung KMY, Whelan M, Willett C, Ankley GT. Harvesting the promise of AOPs: An assessment and recommendations. Sci Total Environ. 2018 Jul 1;628-629:1542-1556. doi: 10.1016/j.scitotenv.2018.02.015. Epub 2018 Feb 22. PMID: 30045572; PMCID: PMC5888775. https://doi.org/10.1016/j.scitotenv.2018.02.015

2. Lee KM, Corley R, Jarabek AM, Kleinstreuer N, Paini A, Stucki AO, Bell S. Advancing New Approach Methodologies (NAMs) for Tobacco Harm Reduction: Synopsis from the 2021 CORESTA SSPT-NAMs Symposium. Toxics. 2022 Dec 6;10(12):760. doi: 10.3390/toxics10120760. https://doi.org/10.3390/toxics10120760

3. Luettich K, Sharma M, Yepiskoposyan H, Breheny D, Lowe FJ. An Adverse Outcome Pathway for Decreased Lung Function Focusing on Mechanisms of Impaired Mucociliary Clearance Following Inhalation Exposure. Front Toxicol. 2021 Dec 14;3:750254. doi: 10.3389/ftox.2021.750254. PMID: 35295103; PMCID: PMC8915806. https://doi.org/10.3389%2Fftox.2021.750254

4. National Academies of Sciences, Engineering, and Medicine. 2017. Using 21st Century Science to Improve Risk-Related Evaluations. Washington, DC: The National Academies Press. https://doi.org/10.17226/24635.

5. National Research Council. 2007. Toxicity Testing in the 21st Century: A Vision and a Strategy. Washington, DC: The National Academies Press. https://doi.org/10.17226/11970.

6. Nersesyan A. 2022. Correct calculations of a "safe" daily dose of raw materials for homeopathic remedies. Re: Toxicological assessment compilation of selected examples of raw materials for homeopathic and anthroposophic medicinal products - Part 2.

Regulatory Toxicology and Pharmacology, 134. Regul Toxicol Pharmacol. 2022 Dec;136:105290. PMID: 36400281. https://doi.org/10.1016/j.yrtph.2022.105290

7. OECD. 2018. "Users' Handbook supplement to the Guidance Document for developing and assessing Adverse Outcome Pathways", OECD Series on Adverse Outcome Pathways, No. 1, OECD Publishing, Paris, https://doi.org/10.1787/5jlv1m9d1g32-en.

8. OECD. 2021. Guideline No. 497 Guideline on Defined Approaches for Skin Sensitisation https://www.oecd-ilibrary.org/fr/environment/guideline-no-497-defined-approaches-on-skin-sensitisation_b92879a4-en

9. Patel, V. S., Amin, K., Wahab, A., Marimoutou, M., Ukishima, L., Alvarez, J., Battle, K., Stucki, A. O., Clippinger, A. J., and Behrsing, H. P. (2023). Cryopreserved Human Precision-cut Lung Slices Provide an Immune Competent Pulmonary Test System for "On-demand" Use and Long-term Cultures. Toxicol Sci doi: 10.1093/toxsci/kfac136. https://doi.org/10.1093/toxsci/kfac136

10. Sharma et al. IN vitro Systems to Predict REspiratory toxicity (INSPiRE): Case study using silanes (In preparation)

11. van der Zalm AJ, Barroso J, Browne P, Casey W, Gordon J, Henry TR, Kleinstreuer NC, Lowit AB, Perron M, Clippinger AJ. A framework for establishing scientific confidence in new approach methodologies. Arch Toxicol. 2022 Nov;96(11):2865-2879. doi: 10.1007/s00204-022-03365-4. Epub 2022 Aug 20. PMID: 35987941; PMCID: PMC9525335. https://doi.org/10.1007/s00204-022-03365-4

12. Varshavsky, J.R., Rayasam, S.D.G., Sass, J.B. et al. Current practice and recommendations for advancing how human variability and susceptibility are considered in chemical risk assessment. Environ Health 21 (Suppl 1), 133 (2023). https://doi.org/10.1186/s12940-022-00940-1
